# Single-virion sequencing of lamivudine-treated HBV populations reveal population evolution dynamics and demographic history

**DOI:** 10.1186/s12864-017-4217-1

**Published:** 2017-10-27

**Authors:** Yuan O. Zhu, Pauline P. K. Aw, Paola Florez de Sessions, Shuzhen Hong, Lee Xian See, Lewis Z. Hong, Andreas Wilm, Chen Hao Li, Stephane Hue, Seng Gee Lim, Niranjan Nagarajan, William F. Burkholder, Martin Hibberd

**Affiliations:** 1 Genome Institute of Singapore, Singapore, 138672 Singapore; 2 Institute of Molecular and Cell Biology, Singapore, 138673 Singapore; 3 London School of Hygiene and Tropical Medicine, London, UK; 4 National University Hospital, Singapore, 119074 Singapore

**Keywords:** Single-virion sequencing, Viral evolution, Adaptation regime, Drug resistance, Chronic hepatitis B, Population demographic history, Bayesian MCMC

## Abstract

**Background:**

Viral populations are complex, dynamic, and fast evolving. The evolution of groups of closely related viruses in a competitive environment is termed quasispecies. To fully understand the role that quasispecies play in viral evolution, characterizing the trajectories of viral genotypes in an evolving population is the key. In particular, long-range haplotype information for thousands of individual viruses is critical; yet generating this information is non-trivial. Popular deep sequencing methods generate relatively short reads that do not preserve linkage information, while third generation sequencing methods have higher error rates that make detection of low frequency mutations a bioinformatics challenge. Here we applied BAsE-Seq, an Illumina-based single-virion sequencing technology, to eight samples from four chronic hepatitis B (CHB) patients – once before antiviral treatment and once after viral rebound due to resistance.

**Results:**

With single-virion sequencing, we obtained 248–8796 single-virion sequences per sample, which allowed us to find evidence for both hard and soft selective sweeps. We were able to reconstruct population demographic history that was independently verified by clinically collected data. We further verified four of the samples independently through PacBio SMRT and Illumina Pooled deep sequencing.

**Conclusions:**

Overall, we showed that single-virion sequencing yields insight into viral evolution and population dynamics in an efficient and high throughput manner. We believe that single-virion sequencing is widely applicable to the study of viral evolution in the context of drug resistance and host adaptation, allows differentiation between soft or hard selective sweeps, and may be useful in the reconstruction of intra-host viral population demographic history.

**Electronic supplementary material:**

The online version of this article (10.1186/s12864-017-4217-1) contains supplementary material, which is available to authorized users.

## Background

Viral intra-host evolution is a critical obstacle in the treatment of chronic infectious diseases. It is the root cause of viral host immune escape and drug resistance, and consequently a major impediment in disease cure and eradication [1, 2]. The hepatitis B virus (HBV) is a prime example. HBV is a small, circular DNA virus. The HBV polymerase is error prone, with an estimated error every 10^5^ to 10^7^ bases [3]. When coupled with a large viral load (often between ~10^3^ copies/ml to 10^7^ copies/ml of serum), this can give rise to substantial viral diversity in active infections [4]. In other words, a sufficiently large viral population can potentially carry, or produce within a short period of time, all possible mutations, thus providing a genetic reservoir for rapid viral response and adaptation [5–10]. Practically, the accumulation of viral mutations is indicative of chronic disease progression and severity [11–13]. Mutations that quickly become predominant in the population are also indicators for how the viruses might be circumventing host response and treatment that enable fresh approaches for drug development research [14].

HBV viral populations, specifically those in chronic infections, can be extremely diverse genetically. Part of the reason is due to high mutation rates leading to the presence of quasispecies [15–20]. Another contributing factor may also stem from genetic repositories in the form of stable covalently closed circular DNA (cccDNA) in infected hepatocytes. Identifying medically important mutations in such populations can become complicated. First, consensus sequence changes occur relatively slowly. For HBV, the mean number of nucleotide substitutions is only estimated at between 1.5 × 10^−5^ to 7.9 × 10^−5^ nucleotide substitutions per site per year [19]. The study of consensus sequences alone may not reveal underlying quasispecies dynamics, which may be much more rapid as the population constantly explores possible genotypes [20]. Second, these hidden quasispecies dynamics may be important in understanding the key indicators of viral fitness. Human host immune response, host genetics, treatment regimes, and finally the viral genotype itself likely interact in a complex fashion that exerts multiple, possibly contradictory selective forces on the virus that ultimately culminates in clinical outcome. Identifying the relevant subpopulation of viruses that are reacting to selective pressures of interest, whether it is nucleoside analogues, interferon treatments, or a change in host immune response can reveal important viral indicators for disease progression.

In order to leverage the recent advancements in next generation sequencing (NGS) technology, we explored single-virion sequencing as an option for characterizing quasispecies diversity in active infections. Deep population sequencing is routinely used to identify polymorphisms, including extremely rare alleles [21–26]. However, without linkage information, it remains difficult to describe quasispecies based on allele frequencies alone. A large number of complete genomes from a single viral population must be sequenced to be confident of full quasispecies diversity. Traditionally, such studies require viruses to be individually cloned and sequenced – a rather tedious process requiring a large amount of work and precious source material [19, 27]. However, the complexity and importance of quasispecies has never been clearer [28], and there are two recent next NGS technologies that can be applied to single-virion sequencing in a high-throughput manner, promising up to thousands of viral sequences from every chronic hepatitis B (CHB) patient sample. BAsE-Seq is an Illumina-based method that makes use of random 20mer barcodes to tag every single viral genome with a unique sequence. The barcoded genomes are then amplified as a single amplicon for library construction [29]. Reads from BAsE-Seq libraries can be reassembled into individual viral genomes in silico post sequencing, effectively constructing thousands of viral genomes with full haplotype information. An alternative approach uses single molecule real time sequencing technology (SMRT) on the Pacific Biosciences platform (PacBio) to produce long reads for individual molecules (up to 60 kb). While single pass sequencing error rates are high, the relatively small 3 kb HBV genomes can be read up to dozens of times by the same polymerase, sharply lowering error rates and yielding highly accurate genome sequences, with the additional benefit of not requiring a reference genome [30].

We aimed to apply these single-virion sequencing methods in a manner tailored to characterizing viral population diversity, quasispecies structure, and population evolution. More specifically, we aimed to discover additional information on viral evolutionary dynamics not visible to regular deep sequencing. We picked a relatively well-understood model – that of HBV resistance to the antiviral drug Lamivudine - where the most common resistance alleles are well characterized [31], and obtained two serum samples from each of four CHB patients who were treated with and subsequently developed resistance to Lamivudine. We searched for resistance mutations in each of the patients and tried to reveal additional quasispecies dynamics using single-virion sequencing. We found that single-virion sequencing reveals vital information about viral population heterogeneity and fluctuations in population composition during viral evolution.

## Methods

### Sample identification and collection in the clinic

Both clonal lab strains and patient samples were used in this study. Plasmids with clonal HBV sequences (referred to as Clone-1 and Clone-2 in the text) were constructed and processed as previously detailed in [28] and sequenced as controls. Patient samples were recruited to test single-virion sequencing on biological populations as well as to describe any additional information that can be gained through haplotype sequencing. Only patients who gained resistance to Lamivudine with serum samples of suitably high viral load (>10^3^ viral copy number/ml) both pre-treatment and post-resistance were considered. As per standard clinical practice, patients who stop responding to anti-viral treatment were tested for resistance mutations through capillary sequencing. For these patients, viral DNA was extracted from 200 μl of serum using the Qiagen Blood mini kit, and the extracted HBV genome was PCR amplified using the Dynazyme DNA polymerase and the primers [Fwd (5′-G[T/C]GTAGACTCGTGGTGGACTTCTCTC-3′).

Rev. (5′-TGACA[T/A/G/C]ACTTTCCAATCA AT-3′)]. The amplified 650 bp fragment was purified by gel electrophoresis and extraction, followed by direct sequencing on an ABI 3730XL DNA Analyzers (SI Table 1). 4 patients with gains of resistance mutations totaling 8 time points had serum samples pulled from the database for sequencing.

**Table 1:** Patient sample nomenclature and viral copy number

Sample ID	Patient	Date	Viral Copies/ul	Single-virion Sequences	Nucleotide Diversity ∏
1.1	1	15th Nov 94	378,500	1717^†^, 1635*	0.0012/base
1.2	1	3rd Jul 99	52,270	3331^†^, 2514*	0.0024/base
2.1	2	22nd May 95	239,750	391^†^, 1330*	0.0032/base
2.2	2	29th Aug 97	44,687	3747^†^, 2504*	0.0013/base
7.1	7	19th May 95	69,180	2647^†^	0.0022/base
7.2	7	11th Apr 00	48,083	789^†^	0.0016/base
11.1	11	19th Jun 95	208,275	2754^†^	0.0211/base
11.2	11	3rd Nov 98	466,200	248^†^	0.0052/base

^†^-total number of single-virion sequences obtained from a BAsE-Seq library. *-total number of single-virion sequences obtained from a PacBio library. Nucleotide diversity Π, the arithmetic mean between all pairwise differences between viral sequences within each viral population, were calculated from BAsE-Seq single-virion sequences for the entire amplified genomic sequence of 3175 bases (3215 minus the 40 bases that were not amplified)

### Barcode-directed assembly for extra-long sequences (BAsE-Seq)

BAsE-Seq was carried out on all samples. They were: Clone-1, Clone-2, Patient 1 timepoints 1.1 and 1.2, Patient 2 timepoints 2.1 and 2.2, Patient 7 timepoints 7.1 and 7.2, and Patient 11 timepoints 11.1 and 11.2. Library preparation was carried out according to the protocol as described in [28]. Briefly, a total of 10^6^ HBV genomes were subjected to a 2-cycle PCR that assigned unique barcodes to each strand of the HBV genome. Two rounds of PCR were carried out to amplify the product, using HBV specific primers (5′-GCTCTTCTTTTTCACCTCTGCCTAATCA-3′ and 5′-GCTCTTCAAAAAGTTGCATGGTGCTGG-3′), taking care to stay within the exponential amplification regime during each round of PCR to minimize the generation of chimeric PCR products [28]. Specifically, a two-stage PCR protocol was employed such that reactions were stopped in the log-linear phase. The final amplicon spans 3175 bp. Samples were exonuclease- digested to generate a pool of nested deletions fragments, which were end-repaired and circularized. Circular products were fragmented and tagged with the Illumina adaptors followed by 14 cycles of PCR to incorporate primers for sequencing. The resulting 2 × 101 bp reads were trimmed for adaptor sequences and base quality with Trimmomatic [32]. A subset of 10,000 read pairs was first BWA-MEM (v 0.7.10) mapped to 8 known HBV genotypes A-H one at a time [33, 34]. All reads were then BWA-MEM mapped to the genotype reference with the lowest number of mismatches. At this point, mapped reads with identical barcodes, signifying their origin from the same viral molecule, are sorted into individual folders for further processing. For each barcode, aligned reads were duplicate-marked, realigned, and recalibrated with GATK v2.7 [35]. Finally, SNVs were called with LoFreq v2.1.2 [36] and incorporated into the final sequences for each barcode (Additional file 1: Figure S4, S5, S8). Full-length viral sequences that passed all quality filters went on to be part of the population analysis [SI]. Maximum Likelihood phylogenies were built from the top 100 sequences with the highest coverage for easier visual interpretation using FastTree v2.1.8 [37, 38]. PHYLIP Neighbor Joining trees were constructed [39] from the full set of viral sequences obtained [SI]. All trees presented were drawn with iTOL [40, 41]. For further details about the pipeline including all processing and error filters refer to [SI].

### Pooled deep sequencing (Illumina)

Pooled deep sequencing was carried out on Clone-1, Clone-2, Patient 1 timepoints 1.1 and 1.2, and Patient 2 timepoints 2.1 and 2.2. Insufficient DNA remained from Patients 7 and patient 11 after BAsE-Seq sequencing, and these four samples had to be excluded from Pooled deep sequencing. For a detailed protocol of sample library preparation, refer to [31]. Briefly, 10^6^ HBV viral genomes were PCR amplified using the same primers as in BAsE-Seq that cover all but the first 40 bp of the 3215 bp genome. 2–3 μg of PCR product for each viral DNA sample was sheared to achieve a fragment size range between 100 and 300 bp. Library preparation was performed using the Qiagen GeneRead DNA Library I Kit according to manufacturer instructions. After end-repair, A-tailing, and adapter ligation, ligated products in the 200–400 bp range were gel-extracted, and subjected to 14 PCR cycles to incorporate multiplexing indices. The final product was quantified and run on a Illumina HiSeq 2000 instrument. Resulting Illumina 2 × 101 bp reads were trimmed by base quality with Trimmomatic and mapped to the concatenated HBV pan-genome consisting of all 8 major genotypes A-H with BWA-MEM (Additional file 1: Figure S1, Table S2). All concordantly mapped read pairs were duplicate-marked, realigned, and recalibrated with GATK 2.7. SNVs present in the pool were called based on comparison with the best match genotype sequence using LoFreq 2.1.2.

### PacBio library construction and analysis

PacBio SMRT sequencing was later carried out on Clone-1, Clone-2, Patient 1 timepoints 1.1 and 1.2, and Patient 2 timepoints 2.1 and 2,2. Insufficient DNA remained from Patients 7 and patient 11 after BAsE-Seq sequencing, and these four samples had to be excluded from PacBio library construction. 10^6^ HBV viral genomes were PCR amplified using the same primers as mentioned above under Pooled Deep Sequencing and BAsE-Seq. 2–3 μg of PCR product was used for PacBio library construction following the 2 kb Template Preparation and Sequencing protocol. Library products were quantified on Agilent 2100 Bioanalyzer, and run on a PacBio instrument with V6 chemistry. PacBio raw reads were first processed with the SMRT Portal analysis programs. To focus on full length functional viruses, circular consensus sequences (CCS) from each library were called with a cutoff of at least 10× subreads within a polymerase read and a minimum subread length of 2500 bp using the RS_ReadsOfInsert application (Additional file 1: Figure 2a, b). CCSs were multiple-sequence aligned against all 8 genotypes with MUSCLE [42]. Bases within the CCS reads with quality scores <75 were masked as Ns to filter out false positives (Additional file 1: Figure. 2c), and the resulting (nearly) full-length viral sequences were BWA-SW mapped as extremely long reads to the concatenated HBV pan-genome consisting of all 8 major genotypes A-H (Additional file 1: Figure 3). (Although a reference panel is not necessary for PacBio long reads, it was included in the analysis here for direct comparison between outputs from the platforms.) Segregating sites within the viral populations were called with LoFreq 2.1.2 with primer regions masked. Maximum Likelihood phylogenies were built from the highest quality 100 CCs sequences of the correct length (3175 bp) using FastTree v2.1.8. Neighbor Joining trees were constructed from the full set of viral sequences obtained using PHYLIP [SI]. All trees presented were drawn with iTOL. For a detailed protocol regarding PacBio read processing and error filters refer to [SI].

### Reconstruction of demographic history by BEAST

A Bayesian Markov Chain Monte Carlo (MCMC) approach was implemented using BEAST v1.8.4 [43] on all sets of 4 patient samples in order to estimate demographic and evolutionary parameters, using the Bayesian skyline plot as a coalescent prior. Unique single-virion sequences constructed from BAsE-Seq libraries often carried missing information due to uneven coverage. Because an excess of ‘N’s can overwhelm the true signal, only the top 100 sequences with the highest overall coverage were used for BEAST analysis. A final fragment of 3134 coding bases was used for demographic history reconstruction. Samples prior to Lamivudine treatment were defined as sequences collected on day 0 and samples post drug resistance annotated as sequences collected *n* days after. We employed the GTR + Γ_4_ unlinked codon model of nucleotide substitution and a strict molecular clock. The MCMC chain length was set to 1E9 to 2E9 generations, depending on the patient sample in question, with sampling of every 1E4^th^. Convergence of the estimates was considered satisfactory when the effective sample size (ESS), calculated in Tracer v1.6, was >200 for all parameters. The first 10% of the estimates was discarded as burn-in. Where necessary, multiple runs were merged using LogCombiner as part of the BEAST package. Run results were analyzed and skyline plots, showing changes in effective population time over time, generated with Tracer v1.6 [44].

### Results

### Single-virion sequencing platform error-rates

**Fig. 1:**
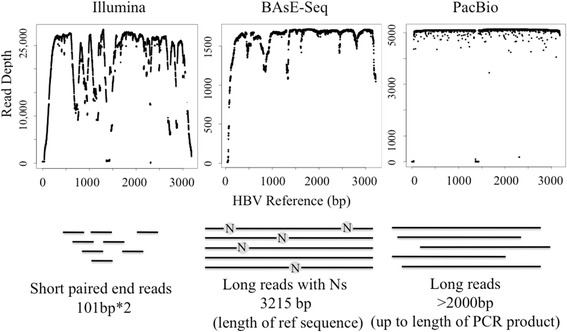
Graphical representation of sequence reads obtained from BAsE-Seq, PacBio, and Pooled Deep sequencing. X-axis: Genome position. Y-axis: Relative coverage. Each dot represents coverage at a single base position. From left to right: Pooled deep sequencing short reads give uneven genomic coverage. BAsE-Seq reconstitutes single-virion sequences by mapping barcoded short reads to a reference, thus matching exact reference length but may have missing information spread throughout depending on local coverage as shown by dips in the coverage plot. PacBio circular consensus reads (CCSs) can vary in length, but will not necessarily require a reference for construction. A library of 3 kb amplicons will cover the entire genome evenly as shown. Dip in coverage around 1.5 kb is due to a deletion in the sample

The three platforms - Pooled Deep sequencing, BAsE-Seq, and PacBio - were tested on two HBV clones with known sequences [28] for pipeline construction and optimization. The data available from each platform is represented in (Fig. 1). Pipelines tailored to each platform were then applied to viral populations from two patients (P1 and P2) to gauge single-virion sequencing performance and throughput on clinical samples [SI]. We began the study with BAsE-Seq, and added PacBio sequencing libraries as the technology became more accessible. We sequenced lab clones with know sequences and estimated the error rates of both methods by summarizing the frequency of base differences in sequenced haplotypes. The base error rates of BAsE-Seq and PacBio libraries were between 0.02–0.3 and 0.2–1.3 per kb of single-virion sequence respectively, and the error rates for small indels in BAsE-Seq and PacBio reads were <0.02/kb and 2.9–3.4/kb respectively. PacBio sequencing is known to have higher error rates in homopolymer runs [45]. We tried to further reduce PacBio error rates through careful selection of PCR polymerase [Additional file 1: Figure S2a-b] and CCS quality filters [Additional file 1: Figure S2c], but still faced multiple sequence alignment issues that gave false positive single nucleotide variants (SNVs) [Additional file 1: Figure S3]. As PacBio errors are random and thus low in frequency when consolidated across all reads, we bypassed this issue by mapping CCs reads to a reference sequence [Additional file 1 Figure S6–S7, S9–S13], and only considering these positions in our population analysis. Because PacBio is a reference independent sequencing technology, it is also possible to map reads to a de novo reference sequence generated from the run. Here, we elected for a common reference sequence across all samples for ease of comparison across BAsE-Seq, PacBio, and Pooled deep sequencing data. A more detailed explanation of all work conducted in this comparison exercise is available in Additional file 1.

### Classic resistance mutations observed

Serum samples were taken from each patient twice for viral DNA library construction - once before they were treated with Lamivudine (labeled as P1.1, P2.1, P7.1, P11.1) and once after viral loads rebounded to detectable levels (labeled as P1.2, P2.2, P7.2, P11.2) (Table 1).

The four libraries from patients P1 and P2 were sequenced on Pooled Deep sequencing, BAsE-Seq, and PacBio platforms. The remaining four libraries from patients P7 and P11 were sequenced and analyzed only by BAsE-Seq due to limited patient serum availability.

**Fig. 2:**
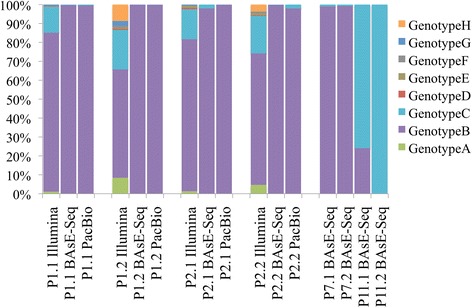
Genotype composition (Y-axis) of samples sequenced (X-axis) as reported by BWA-SW coverage across 8 reference genotype sequences. Samples are organized from left to right in order of P1.1, P1.2, P2.1, P2.2, P7.1, P7.2, P11.1, and P11.2, where P1.1 and P1.2 are two time points from the same patient P1, before and after drug resistance respectively. Wherever data from multiple technologies are available, order follows Pooled deep sequencing, BAsE-Seq, and lastly PacBio

Viral genotype composition in each sample was estimated from the percentage of reads mapping to each genotype reference in the pan-genome panel. Three out of four patients carried Genotype B viruses. The only exception was P11, who carried a mixed Genotype B and Genotype C infection prior to drug treatment, but only Genotype C viruses post Lamivudine resistance (Fig. 2). Illumina short reads tend to mis-map in regions where sequence divergence is ~3% between the references used, an issue absent in BAsE-Seq and PacBio long reads [Additional file 1 Figure S4]. Therefore, genotype identification in Illumina libraries must take into account evenness of coverage across the references, or number of mismatches in mapped reads, in addition to absolute percentage of reads mapped.

Lamivudine resistance is achieved through mutations in the reverse transcriptase (RT) domain of the polymerase gene in HBV [46–48]. Two resistance phenotypes made up of three amino acid changes, M204I and L180 M + M204 V, are the most commonly observed. They confer similarly high resistance and only require one to two nucleotide changes [49]. Both of these resistant genotypes were found, and together explained resistance in all four patients (Table 2). The discrepancy in allele frequencies between the platforms may have been due to sampling error of low viral load samples.

**Table 2:** The frequencies of detected resistance alleles in each of the 4 patients after drug resistance. None of these mutations were observed (below detection limit) in the populations prior to development of resistance

#	Mutation	Pooled Deep Seq	BAsE-Seq	PacBio
P1	M204 V L180 M M204I	0.525 0.560 0.457	0.711 0.752 0.251	0.567 0.611 0.358
P2	M204I	0.978	0.882	0.990
P7	M204 V L180 M		0.870 0.946	
P11	M204I L180 M		0.948 0.679	

**Fig. 3:**
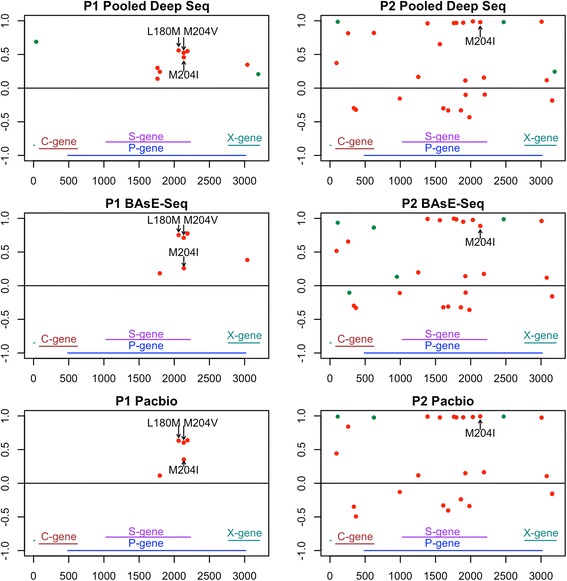
Change in allele frequencies of variable sites in P1 and P2 after drug treatment (detection limit >0.01). Left panels shows the delta change in allele frequencies of segregating sites (Y-axis) across the genome (X-axis) in patient 1 on all 3 sequencing platforms. Right panels show the corresponding data for patient 2. All non-synonymous changes were colored in red, synonymous changes in green

**Fig. 4:**
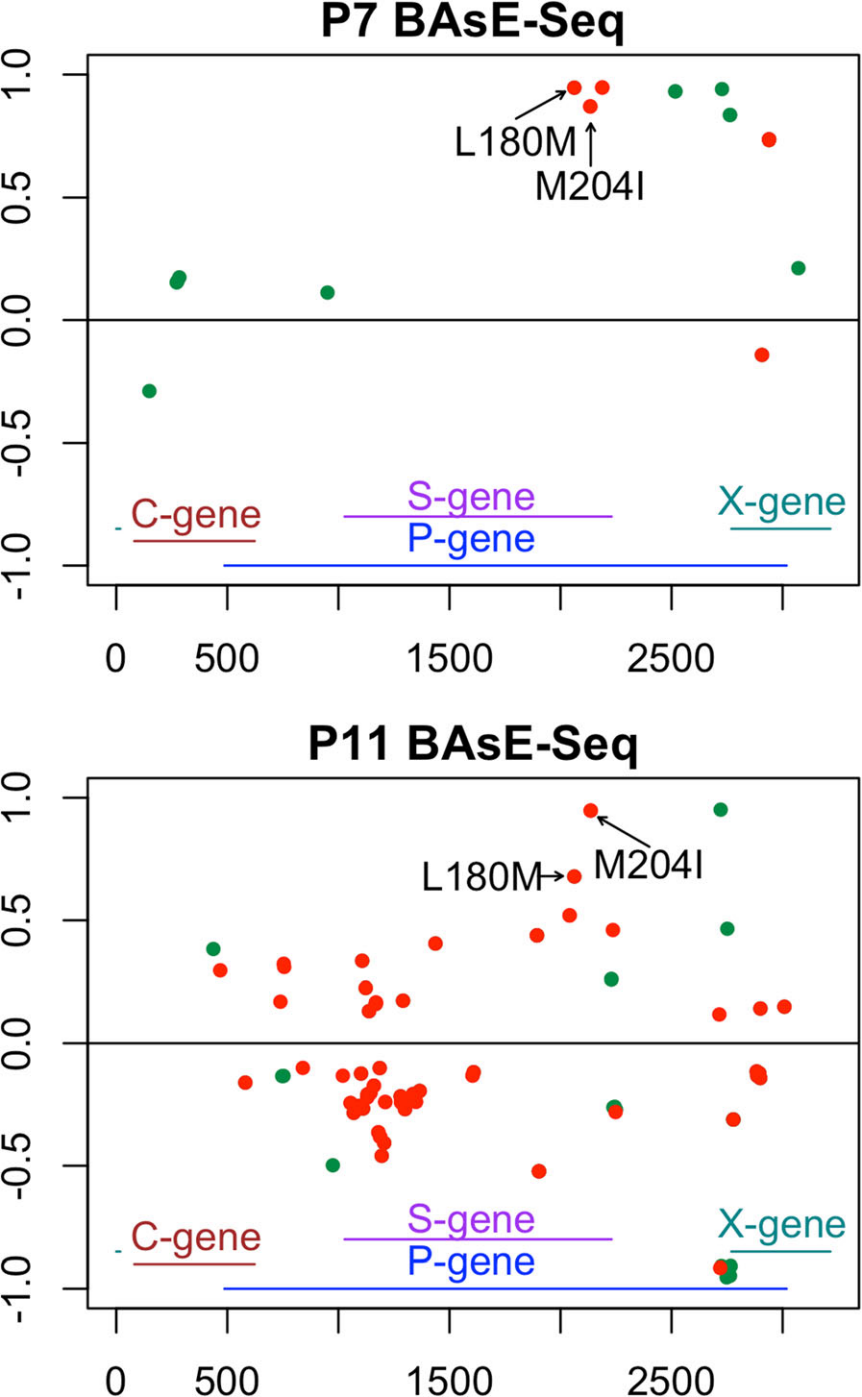
Change in allele frequencies of variable sites in P7 and P11 after drug treatment (detection limit >0.01). Panels show the delta change in allele frequencies of segregating sites (Y-axis) across the genome (X-axis) from BAsE-Seq libraries. All non-synonymous changes were colored in red, synonymous changes in green

While P2 (M204I) and P7 (M204 V+ L180 M) carried single resistance phenotypes, P1 carried both M204I and M204 V+ L180 M. The genotypes are not mutually exclusive and all three of the point mutations were found in the same patient at significant frequencies (Figs. 3, 4). P11 was nearly fixed for M204I, but also carried L180 M at a high frequency.

## Discussion

### Viral Quasispecies reveal both hard and soft selective sweeps

Single-virion haplotypes should yield deeper insights into how these resistance genotypes evolved. Here, we asked if we could identify whether resistance mutations came from a single source and quickly swept to high frequency (hard sweep), or multiple sources that then grew in frequency independently (soft sweep) [50–53]. Note that this question is extremely difficult to address without long-range haplotype information. Hard sweeps are likely to happen with lower mutation rate or extremely strong selection, where adaptive mutations occur one at a time and immediately outcompete other genotypes within the population. Soft sweeps tend to dominate if mutation rates are higher, selection is milder, population is large [54], and multiple lineages carrying advantageous mutations may be present at a time, all increasing in frequency due to the consequent selective advantage [55, 56]. While HBV is a DNA virus that mutates relatively slowly as compared to RNA viruses, it is also true that Lamivudine exerts a strong selective pressure against viral replication. We also asked if we could identify whether resistance alleles were from de novo mutations or from existing low frequency variants. Adaptation from de novo mutation is usually defined as serial fixation of novel alleles, with just one adaptive allele rising to fixation at a time, whereas adaptation from standing variation often also carries with it multiple pre-existing mutations linked to the advantageous allele [57–60]. Which model is more relevant is partly determined by population diversity and the presence of pre-existing drug resistant strains. A clonal viral infection, such as a recent or mono-strain infection seeded by very few drug-naïve virions is less likely to carry pre-existing resistance alleles as compared to a mixed infection or a long-term infection that has had time to diversify within the patient. There is also the possibility that a large, highly mutable viral population could theoretically carry all possible mutations in its quasispecies mutant pool at any point in time. Determining the correct model for HBV population evolution will be important for describing and modeling adaptation.

We made use of nearly full genome haplotype information from BAsE-Seq to characterize viral population quasi-species composition before and after drug treatment (Additional file 2). PacBio trees for P1 and P2 are available in SI. Phylogenetic trees built from viral haplotypes revealed three different patterns in how these patients gained viral resistance (Additional file 3).

**Fig. 5:**
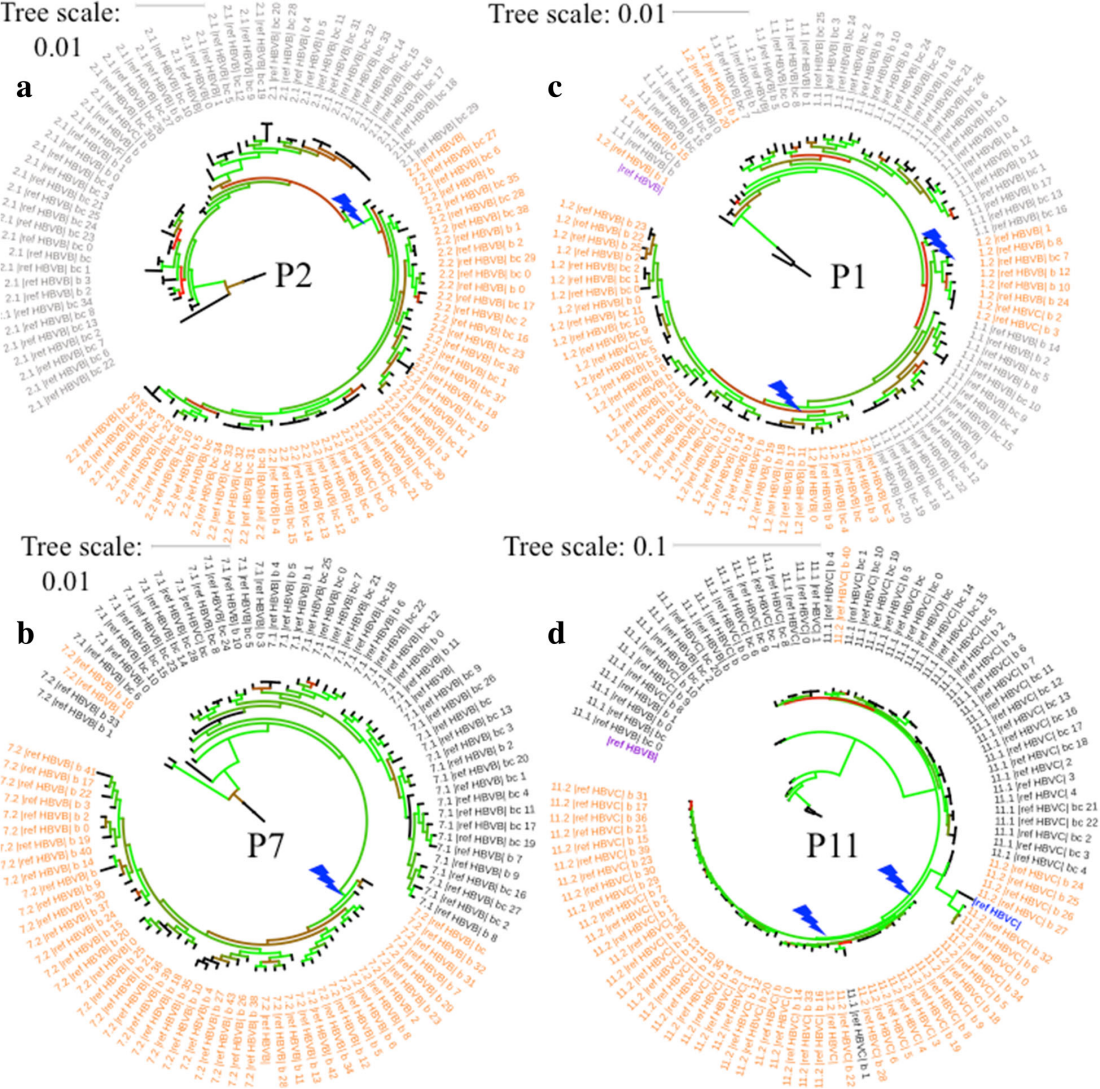
BAsE-Seq approximately maximum-likelihood trees of viral sequences from P2 (a), P7 (b), P1 (c), and P11 (d). Tip label colors indicate if the viral sequence came from before (black) or after (orange) drug resistance. 100 sub-sampled sequences from each timepoint are used, color-coded by their branch labels. Bootstrap values are reflected in branch color, ranging between 0 (red) to 1 (green). Blue arrows indicate emergence of resistance mutations. For a full phylogeny of all sequences, refer to (Additional file 1 Figures 14–17)

**Fig. 6:**
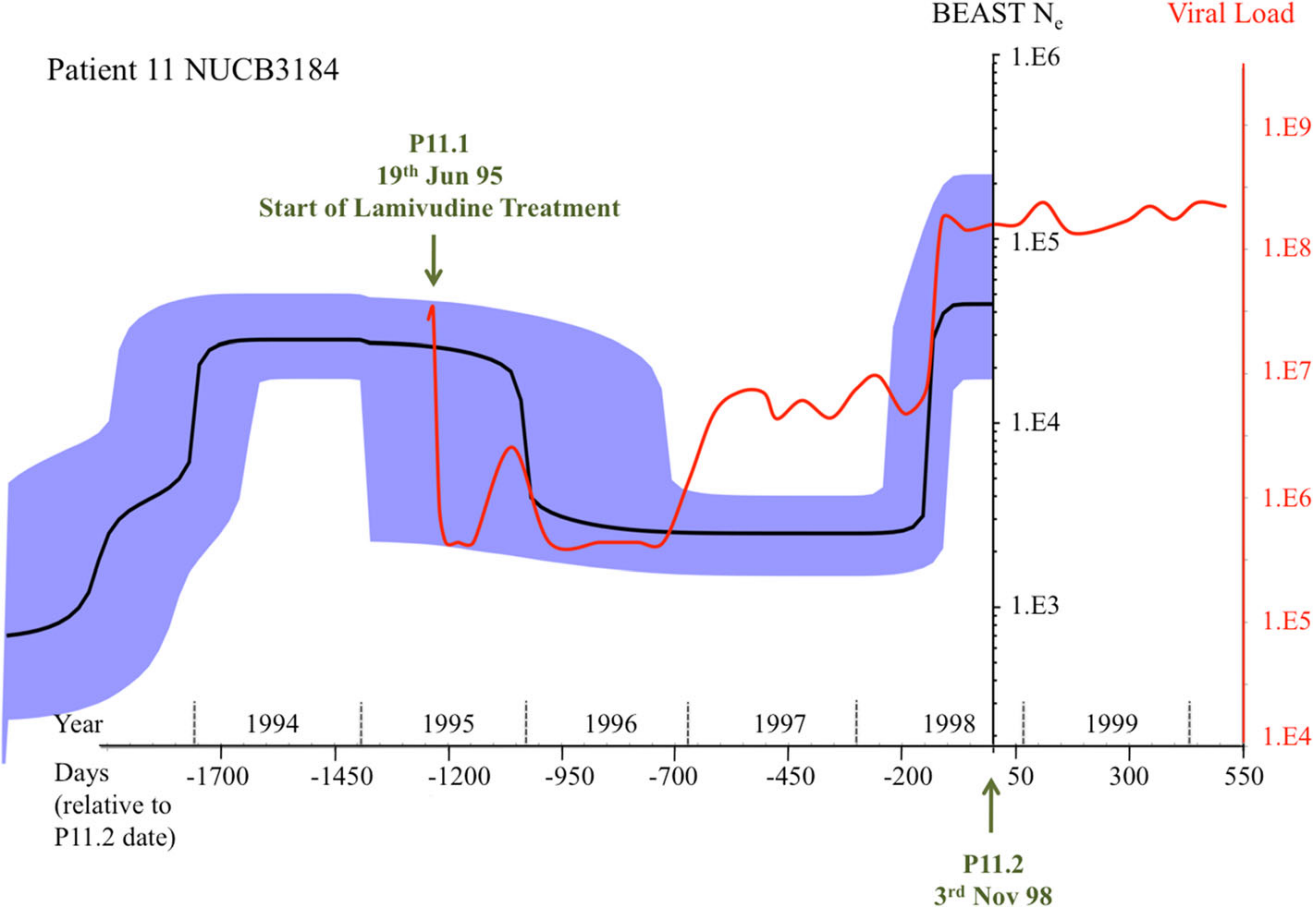
Overlay of BEAST reconstructed demographic history and clinical records of patient viral load (serum) for patient 11. X-axis – Timeline represented in days, going forward in time from left to right. Green arrows point out the two timepoints that were used for single-virion sequencing. Y-axis labeled “Viral load” – corresponding to red line tracing all available records of patient viral load (log10 scale) over time. Y-axis labeled “BEAST N_e_” - Effective population size over time as simulated by BEAST (log10 scale) shown by black line (plotted values are local medians, with 95% highest probability density (HPD) confidence interval colored in blue)

Two patients, P2 and P7, had trees that showed clear mono-clonal gains of resistance, suggestive of hard sweeps (Fig. 5a,b, Additional file 1 Figure S14–S15). Allele frequency changes showed clusters of SNVs that increased in allele frequency together (11 SNVs in P2 spanning the entire 3.2 kb sequenced region [Fig. 3] and 6 SNVs in P7 spanning 2 kb–2.8 kb [Fig. 4]). Haplotype information confirmed that these were linked SNVs on the same haplotype. 9/11 SNVs in the P2 cluster were within the RT domain of the polymerase gene, and 7/11 SNVs were non-synonymous mutations within a 750 bp window. All six SNVs in the P7 cluster were within the RT domain of the polymerase gene, and three were non-synonymous mutations within a 150 bp window. These two sweeps with numerous SNVs linked to the resistance allele would support a model of evolution from standing variation. However, these exact combinations of SNVs were not found in the treatment naïve timepoints for either patient. The closest haplotypes found pre-treatment shared just 6/11 SNVs for P2 and 2/6 SNVs for P7. Haplotype analysis of linked SNVs in PacBio sequences for P2 showed the same pattern (Fig. 3).

We suggest three possible explanations for this linkage. First, there could be a detection limit for extremely rare haplotypes in the pre-treatment timepoints. We may simply have failed to sequence them. Second, because our samples came from patient blood samples, latent viral reservoirs outside of the blood stream could be contributing to the viral population. Perhaps even reshuffling viral haplotypes through recombination. Again, they would be missed by serum samples. Finally, the resistance mutation could have occurred later during the treatment regime by chance, and happened to rise on the background of a viral sequence that already accumulated multiple nucleotide differences from the population consensus. There was in fact a two to three fold difference in nucleotide diversity across the eight patient samples (Table 1), suggesting a range of quasispecies complexity across patients, which may affect observed evolutionary dynamics.

The two remaining patients, P1 and P11, had trees showing at least two independent instances of gain of resistance, in other words soft sweeps (Fig. 5c,d, SI Additional file 1 Figure S16–S17). P1 was highly clonal with just 6 sites shifting in frequency over time (Fig. 3), and independently gained M204I and L180 M + M204 V on two haplotypes. Again, this was seen in the PacBio library as well (Fig. 3). P11 carried the most diverse population out of all four patients, starting as a mixed population of 26% Genotype B and 74% Genotype C (Fig. 2). The same resistance allele M204I evolved twice on Genotype C sequences but none on Genotype B sequences, resulting in a resistant viral population that was 100% Genotype C. One lineage further gained the L180 M mutation, although it is unclear whether that conferred additional resistance on a M204I background.

#### Reconstructing demographic history

The BEAST analysis for P11 showed effective samples sizes (ESS) of >500, indicating convergence. Sequences came from day −1233 (sample before Lamivudine treatment) and day 0 (sample taken after viral resistance and consequently viral load rebound) [SI Table 3]. Reconstructed demographic history showed an initial exponential growth phase post infection, followed by a plateau. A sharp dip in effective viral population size (N_e_) then occurred sometime between −1350 to −750 days prior to day 0. From actual patient viral load information, the population crash post drug treatment occurred right after the sample was take on day −1233. There was also a sharp increase in median N_e_ about 150 days prior to day 0 in the skyline plot, which matched clinical data almost perfectly (Fig. 6). Although the two major changes in population size were well identified, a smaller increase in the viral population size around day −700 was not, possibly indicating some limitations when reconstructing smaller scale changes in population demography, or that these viruses did not contribute to the effective population size. Due to the smaller nucleotide diversity present in the other patient samples (Table 1), runs for P1, P2 and P7 did not coalesce at 1E9 replications (Additional file 1 Figure S18–S20).

## Conclusions

Haplotype information is vital for revealing hidden population dynamics invisible in standard deep sequencing data. While single-virion sequencing remains technically challenging, we employed two complementary single-virion sequencing platforms to reveal – and cross-validate - such information. We can tell, from nucleotide diversity calculations, the heterogeneity of a population. We can estimate, using up to thousands of single-virion haplotypes, the relative proportions of genotypes and quasispecies present in an infection. We can determine if resistance evolved from a single source, or multiple times independently. Using samples taken at different timepoints, we can begin to explore whether evolution occurs from standing variation or de novo mutations, and how that is linked to quasispecies complexity. While lamivudine resistance is a relatively simple adaptive process with very specific alleles conferring fitness gains, this work shows the potential of applying single-virion sequencing to complex events such as viral response to immune enhancement or viral dynamics during an active HBV flare. It may also be valuable in the study of difficult topics such as cccDNA stability, viral recombination, and viral reservoirs. Single-virion sequencing is therefore a powerful tool for understanding the role of viruses across disease stages of clinical importance.

## Additional files

Additional file 1: A supplementary materials file provides additional technical details and figures deemed unnecessary for the main text, including BEAST results for all patient samples. (PDF 2754 kb)

Additional file 2: Supplementary_genomes.fasta. High quality single virion sequences Single-virion sequences that were reconstructed with BAsE-Seq and used in FastTree phylogeny analysis for all 4 patients. (FASTA 1289 kb)

Additional file 3: Supplementary_FastTrees.txt. Newick format phylogenetic trees. FastTree output in Newick format for all 4 patients (TXT 16 kb)

## Abbreviations

BAsE-Seq: Barcode-directed Assembly for Extra-long Sequences
cccDNA: covalently close circular DNA
CCs: circular consensus sequence
CHB: chronic hepatitis B
ESS: effective sample size
HBV: hepatitis B virus
MCMC: Markov chain Monte Carlo
NGS: next generation sequencing
SMRT: Single Molecule, Real-Time
SNV: single nucleotide variant.
