# Supplementary material for: Single-virion sequencing of lamivudine-treated HBV populations reveal population evolution dynamics and demographic history
Source: BMC Genomics. 2017 Oct 27;18:829. doi: 10.1186/s12864-017-4217-1 (PMC5660452; doi:10.1186/s12864-017-4217-1)
Supplement: Supplementary file 1 — A supplementary materials file provides additional technical details and figures deemed unnecessary for the main text, including BEAST results for all patient samples. (PDF 2754 kb) [file 12864_2017_4217_MOESM1_ESM.pdf]

Single Virion Sequencing of Lamivudine Treated HBV Populations
Reveal Hidden Population Evolution Dynamics

Supplementary Materials

Identifying Patient Samples

RESISTANCE MUTATION TESTING

Patient Number	RT-80	RT-91	RT-109	RT-118	RT-121	RT-122	RT-124	RT-127	RT-131	RT-134	RT-145	RT-151	RT-180	RT-204	RT-207	RT-221	RT-222	RT-224	RT-238	RT-264
	L	L	S	N	I	I	N	R	N	N	M	Y	L	M	V	Y	T	V	H	L
P1.1																	A			
P1.2													M	V						
P2.1																				
P2.2	V	I					D							I						R
P7.1															L		A			
P7.2													M	V	L					
P11.1			P	T	N		Y		D	D	L	F				F		I	N	
P11.2	V		P	T	N		Y		D	D	L	I	M	I		F		I	N	R

aa changes in RED

SI Table 1. Mutations identified in Lamivudine resistant patients as by 3730XL DNA analyzer. All amino acids are represented by alphabets as follows (V-Val, I-Ile, P-Pro, T-Thr, N-Asn, Y-Tyr, D-Asp, L-Leu, F-Phe, M-Met, A-Ala, R-Arg). Each column represents an amino acid position on the reverse transcriptase gene (RT). 2nd row shows reference sequence amino acids in black (genotype B). Each following row represents a single patient time point, with two temporal time points per patient. Amino acid changes as compared to the reference genome are represented in red; resistance mutations are highlighted in gray.

Picking Reference Genomes

There are at least 8 well-characterized HBV genotypes with up to 10% pairwise sequence difference (Sunbul 2014). Rather than mapping all patient samples to a single sequence and identify viral genotype post-hoc, and to include some flexibility for the identification of mixed-genotype infections, one complete sequence from each of genotypes A through H was compiled into a species 'pan-genome' [SI Fig 1]. Longest published sequences that were the most recently published were selected. The final sequences used came from HB-JI444AF (genotype A, Genbank: AP007263.1), P2-121214 (genotype B, Genbank: AB981583.1), P1-090725 (genotype C, Genbank: AB981580.1), MK096_EP-CHB (genotype D, Genbank: KM524346.1), Mart-B84 (genotype E, Genbank: HE974384.1), HBV-BL592 (genotype F, Genbank: AB166850.1), CLB-DonX (genotype G, Genbank: GU565217.1), and B-MHJ9014 (genotype H, Genbank: AB846650.1). Because the HBV genome is circular, not all published sequences start and end at the same position along the genome. Published sequences were reassigned base positions such that they begin and end in positions complimentary to our PCR primer products (SI Figure 1). A multi-sequence alignment of the 8 genotypes revealed a 6bp insertion in genotype A, a 33bp deletion in genotype D, a

3bp deletion in genotype E, and a 36bp insertion in genotype G. An aligned fasta file with all 8 genotypes is appended.

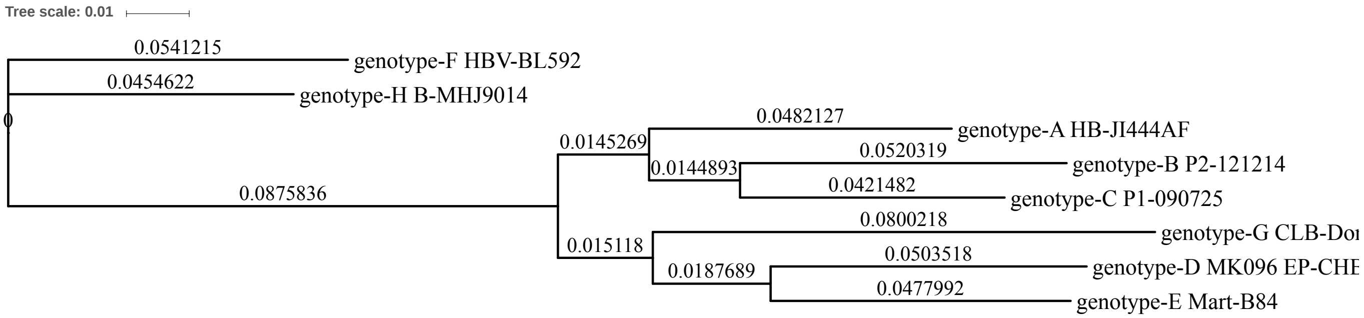

SI Figure 1. Phylogenetic tree of eight reference genomes used in the pan-reference.

The pairwise sequence identity between the genotypes ranged between 85%-92%, as shown below (SI Table 2).

	A_JI444AF	B_P2-121214	C_P1-090725	D_MK096_EP-CHB	E_Mart-B84	F_HBV-BL592	G_CLB-DonX	H_B-MHJ9014
A_JI444AF	100%	90%	92%	90%	89%	86%	89%	86%
B_P2-121214	90%	100%	91%	89%	88%	85%	87%	86%
C_P1-090725	92%	91%	100%	90%	89%	86%	88%	86%
D_MK096_EP-CHB	90%	89%	90%	100%	92%	86%	89%	86%
E_Mart-B84	89%	88%	89%	92%	100%	85%	89%	85%
F_HBV-BL592	86%	85%	86%	86%	85%	100%	85%	92%
G_CLB-DonX	89%	87%	88%	89%	89%	85%	100%	85%
H_B-MHJ9014	86%	86%	86%	86%	85%	92%	85%	100%

SI Table 2. Pairwise sequence identity between eight reference genomes used in the pan-reference.

PacBio Analysis Pipeline Development

PacBio raw reads were first processed with the SMRT Portal in house analysis programs. Because single pass reads have base error rates averaging $1.4x10^{-2}$/base, circular consensus sequences (CCS) from each library were called with a stringent cutoff of at least 10x subreads within a polymerase read and a minimum subread length of 2500bp (to retrieve full functional genomes). Theoretically, this lowers base error to $2.89x10^{-19}$/base. While the required number of passed required to call a CCS can be adjusted, we picked 10x to maximize quality while maintaining a minimum level of usable data (SI Figure 2).

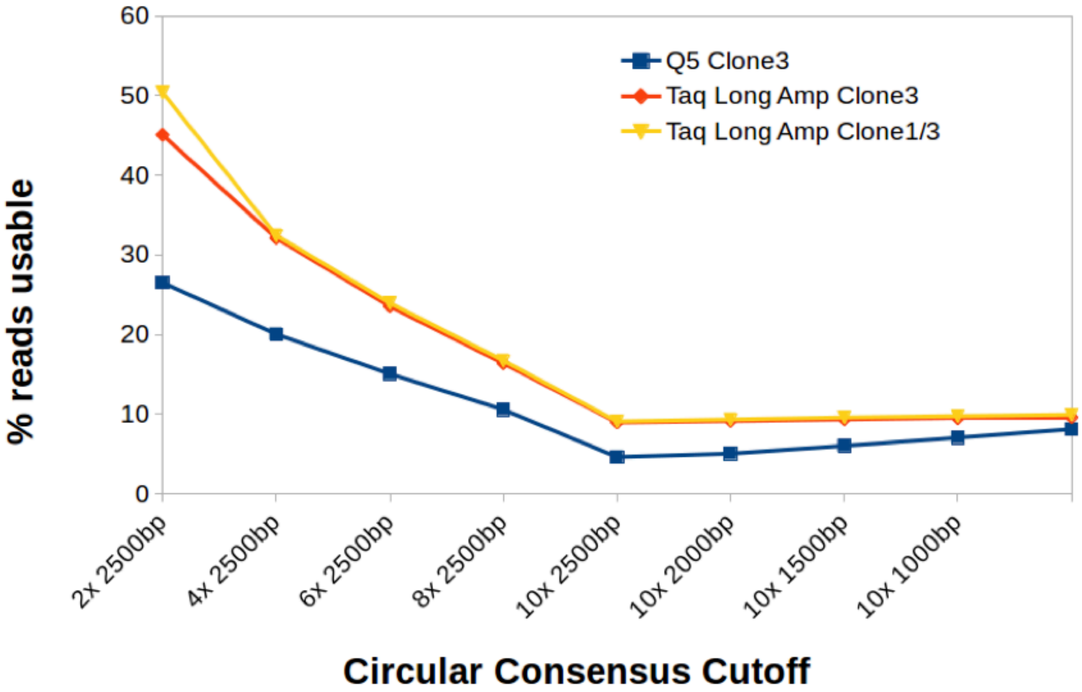

SI Figure 2a. Relation between number of passes and read length required to call a CCS (X-axis) and the % of reads usable from a cell of PacBio (Y-axis). Data shown comes from three test libraries constructed with templates made from two different long-range PCR polymerases, Q5 and Taq Long Amp.

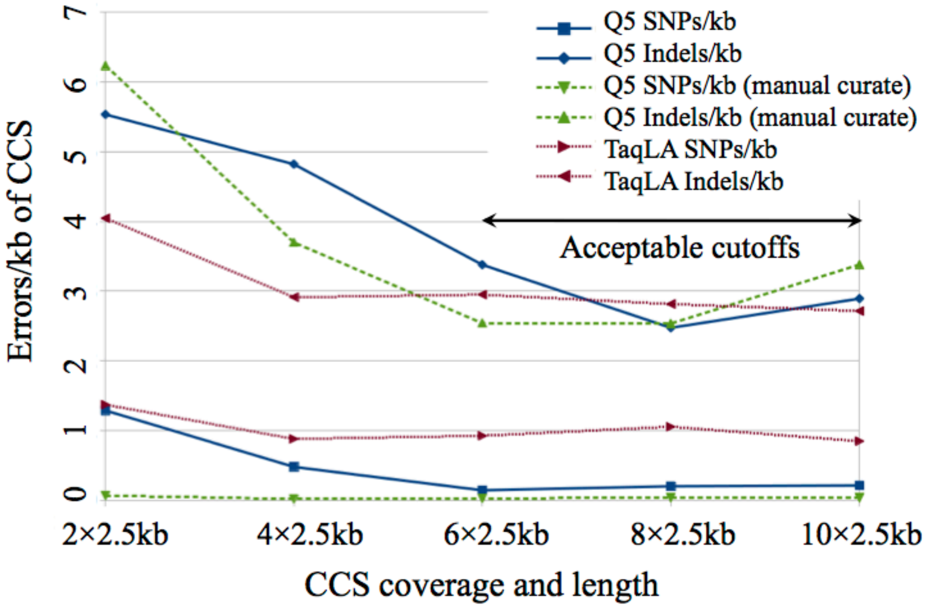

SI Figure 2b. Relation between number of passes and read length required to call a CCS (X-axis) and the error rate per kb (Y-axis). Data shown comes from two test libraries constructed with templates made from two different long-range PCR polymerases, Q5 and Taq Long Amp, and manual curation of Q5 reads.

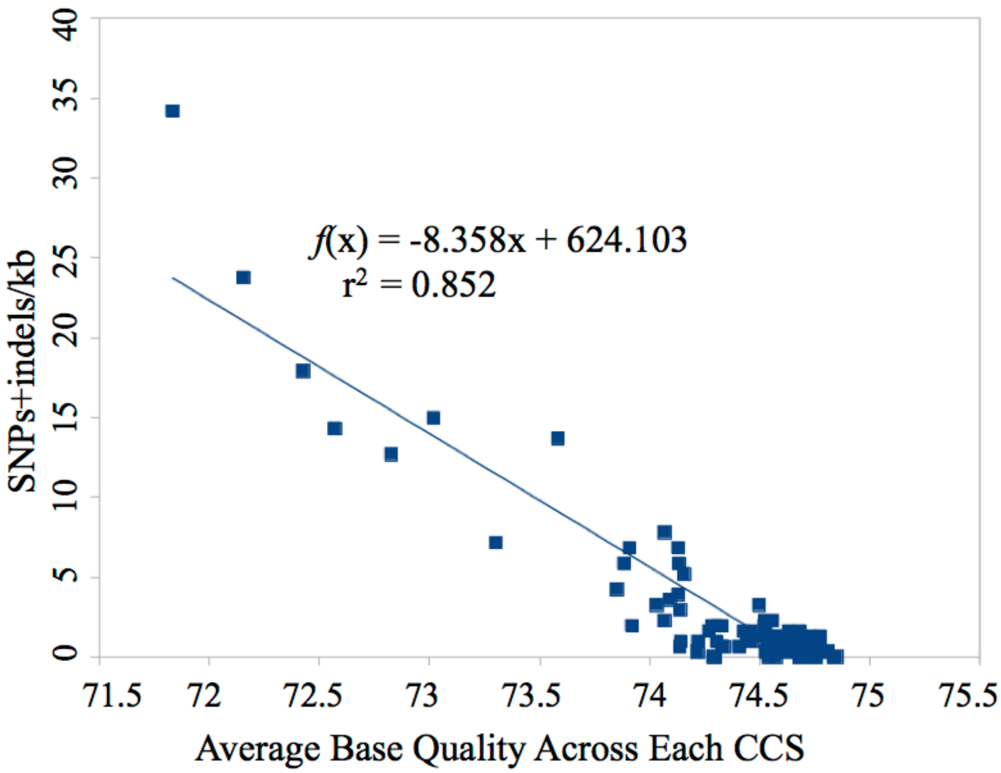

SI Figure 2c. Data shown comes from a PacBio sequencing run of a known clone. Graph plots correlation between 10x CCS base quality (X-axis), averaged across all bases in the CCS, and the number of SNPs and small indel errors observed per read as compared to known clone sequence (Y-axis).

Bases within the CCS reads with quality scores <75 were masked as Ns so as to filter out false positive SNPs, and the resulting (nearly) full viral sequences were BWA-SW mapped as extremely long reads to the concatenated HBV pan-genome consisting of all 8 major genotypes A-H. It is worth noting that because small indel errors were still present at an estimated rate of ~3/kb (SI Figure 3), small indels were not called in our analysis. Segregating sites within the viral populations were called with LoFreq.

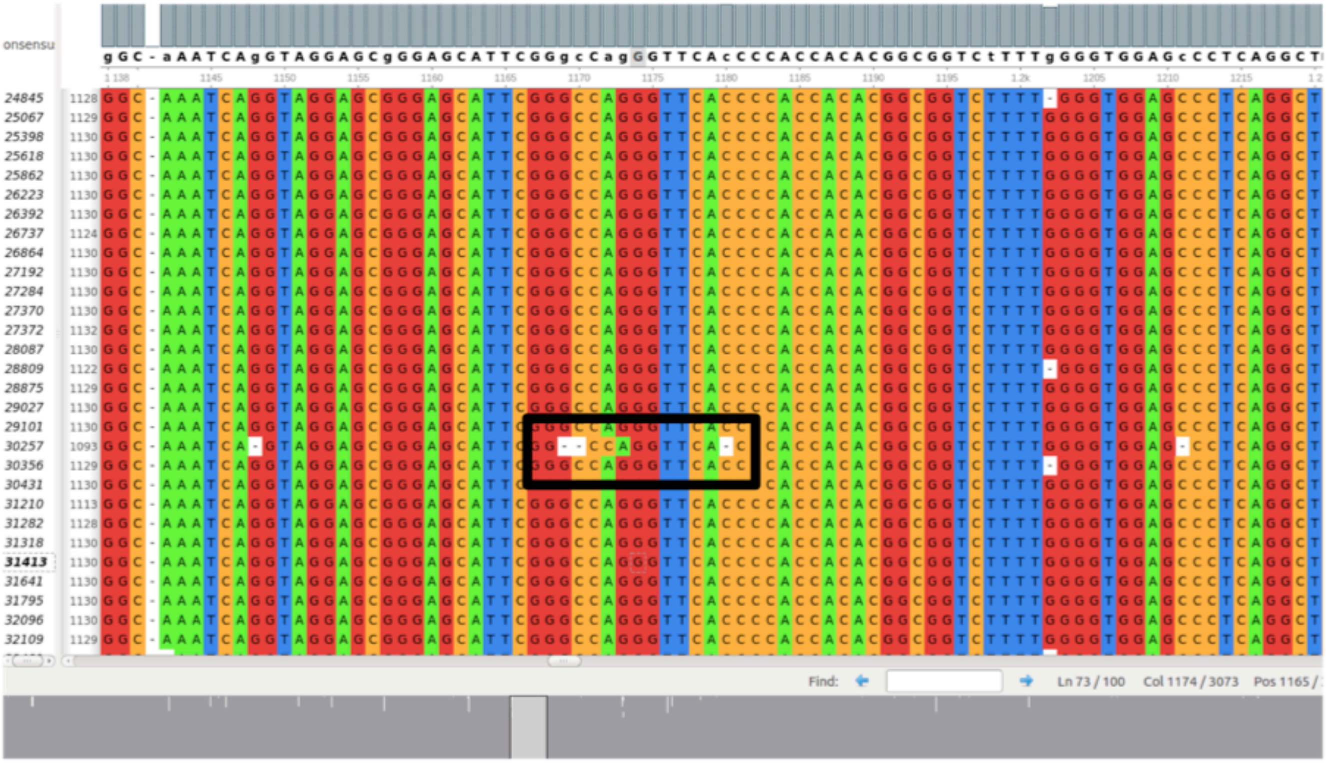

SI Figure 3. A screen cap of the small indel errors seen in PacBio 10x CCS reads.

BAsE-Seq Analysis Pipeline Development

BAsE-Seq 2×150bp reads (sequenced on a Mi-Seq machine) were processed with a custom pipeline modified from a previous publication (Hong et al., 2014). To remove unique barcodes and primer contamination, fastq reads were first trimmed by adaptor sequences and base quality >30. Resulting fragments were filtered for read pairs with both reads passing the minimum length requirement of 20bp. A subset of 1,000 trimmed read pairs were mapped to each of the 8 major genotypes A-H independently. The best match genotype was identified by lowest average mismatches, and all reads were BWA-MEM remapped to this single genotype sequence. This was done such that mapping errors could be minimized as much as possible. All concordantly mapped read pairs were sorted into individual sam files by their unique barcodes, identifying them as reads from the same viral genome. Each sam file was duplicate-marked, realigned, recalibrated, and SNPs were called with LoFreq for incorporation into the viral sequence. (At this stage, an additional step can be added for mixed genotype infections. Sam files with excessive mismatches can be remapped to next best match reference if necessary.) Because each sam file represents data from a single viral genome, SNPs were called if coverage depth >4x and 100% of reads supported the alternative allele. All bases with coverage depth <4x and/or <100% of reads supporting the same allele were marked as "N". This step was aimed at minimizing false positives at error prone regions. Average genome coverage and read depth across all individual sam files were tested across a range for best cutoff to select an optimum number of usable sequences (<50% "N"s) for subsequent analysis. Viral sequences that passed the quality filters were written into an original sam

files as long reads that map to position 1 of the reference. LoFreq was used to call segregating sites within the population.

LoFreq Variant Calling

Variants were called with LoFreq in Pooled deep sequencing and PacBio using default filters.

In BAsE-Seq, LoFreq was employed thrice. The first time, LoFreq was used with default settings on the sam file containing all reads (before assignment into individual sam files by barcode). This step was found to be necessary for marking positions with strand bias error that will lead to false positives in the individual genomes. The second time, during the variant calling in individual genomes, coverage was often too low to accurately detect strand bias and will lead to false positives. Positions prone to strand bias errors as previously noted are ignored in this step. Finally, LoFreq was used on all of the reconstructed single virion sequences with the option --no-default-filter to turn off the filter for strand bias. BAsE-Seq long reads are built from the reference and thus artificially single stranded.

In addition, LoFreq considers Ns as covered bases (although they are not used in actual variant calling). For BAsE-Seq and PacBio libraries where Ns were introduced to mask low quality or low confidence bases, LoFreq output was parsed to re-calculate the actual allele frequencies of SNPs without Ns.

At the final stage, all SNPs called by LoFreq were further filtered by allele frequencies >0.01 and quality score >1,000 to remove false positives.

Picking Test Populations

In order to compare BAsE-Seq against Pooled deep sequencing and PacBio SMRT sequencing, we used two clones with known sequences on all three platforms. We selected the same Clone-1 and Clone-2 strains that were previously used in BAsE-Seq development (Hong et al., 2014). Clone-1 and Clone-2 are both Genotype C isolates, with 17 base differences between them. When compared against the Genotype C sequence used in our mapping reference, Clone-1 has 56 nucleotide changes (1.7% sequence divergence) and a 184 base pair deletion inclusive of bases 1197-1379. Clone-2 has 60 (1.9% sequence divergence) nucleotide changes and a 66 base pair deletion inclusive of bases 1380-1445.

Homogeneous Viral Population - Clone-2.

We sequenced Clone-2 singly by BAsE-Seq, Pooled deep sequencing short reads, and PacBio to estimate variant detection error rates and gauge best quality filters moving forward. PCR amplified Clone-2 templates were constructed into libraries for each of the three platforms. For Pooled deep sequencing, Clone 2 library was multiplexed as 1/55 of a lane using the HiSeq SBS kit v4 in high output mode, with paired-end 2x125bp reads. When mapped to the reference panel, fastq reads gave an average coverage depth of ~23,000x within genotype C. Two regions of the genotype C sequence were expected to be missing from fastq reads - bases 0-40 were not amplified during PCR, and bases 1380-1445 contained a known deletion. Both these regions had coverage depths below 1000x (<5% average genomic coverage) (SI Figure 4).

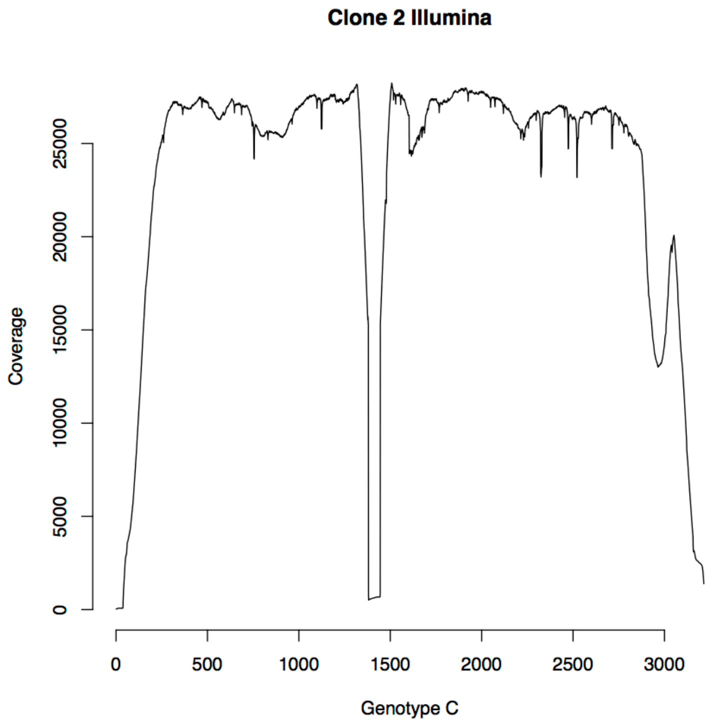

SI Figure 4. Coverage (Y-axis) plot against Genotype C (X-axis) for Clone 2 Pooled deep sequencing library. The first 40bp were not covered due to primer location, and a 66bp deletion is also missing coverage starting at position 1380.

Outside of the Genotype C region, there was also a short aberrant peak in the last 300bp of genotype E (SI Figure 5 top panel).

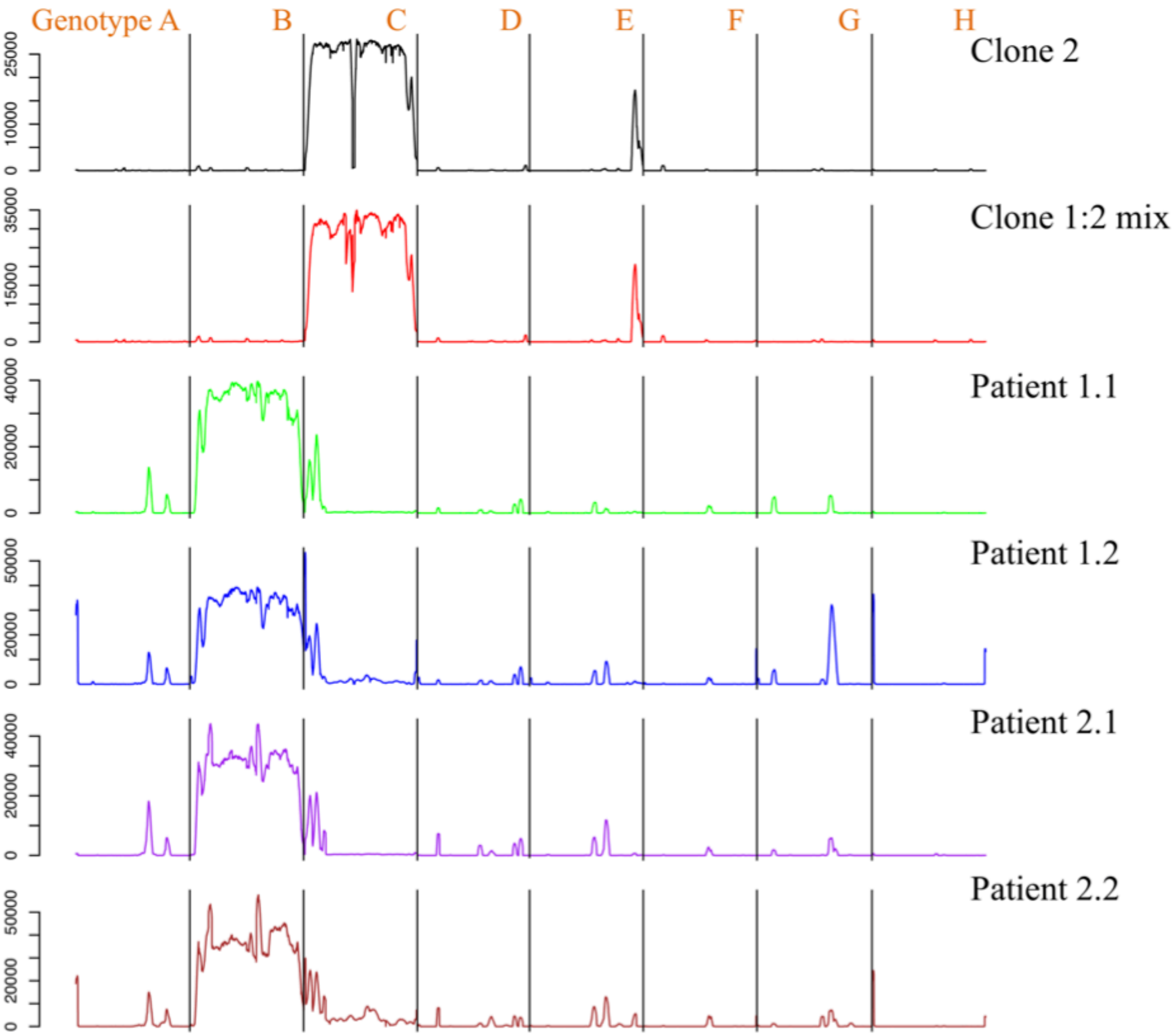

SI Figure 5. Coverage (Y-axis) plot against Genotype A-H pan-genome (X-axis) for all six Pooled deep sequencing libraries. Vertical lines demarcate where the 8 genotypes begin and end. Clone 2 and Clone 1:2 mix are Genotype C clones, while the lower four patient samples are Genotype B infections. Some mismapping peaks can be seen outside of the main genotype due to localized sequence similarity.

This region was 3.5% divergent from genotype C, and was presumed to be prone to mis-mapping due to high sequence similarity. Excluding 3 nucleotide differences present in bases 0-40, all other 57 known SNPs were accurately called. 7 false positives SNPs were also called, 4 in low coverage regions and 3 due to strand bias. We found that a SNP quality cutoff of 1000, a coverage cutoff of >5% average (within the best match genotype), and an allelic frequency cutoff of 1% were required to minimize false positives.

The Clone 2 BAsE-Seq library yielded consensus sequences for 8,796 viral genomes with >1500 bases (~50% of genome) covered to >4x read depth. Insertions could be theoretically called for each sequence but are usually low confidence due to the low coverage; therefore indels were not part of the pipeline and were not incorporated

into the sequences. Known deletion regions showed no coverage across all reconstructed sequences, which is a possible clue for identifying novel deletions. 2 SNPs that were strand bias induced false positives were subsequently removed. All 57 SNPs expected were correctly identified with a SNP quality cutoff of 1000 and an allelic frequency cutoff of 1%.

On PacBio, Clone 2 library was run on a full cell (v6). All 5,110 CCSs were BWA-SW mapped as extremely long reads to the reference panel. Four CCSs (0.08%) were mis-mapped onto incorrect references – two mapped to genotype E due to large numbers of low quality bases (>50% of read), and the remaining two mapped to genotype D due to sequence chimerism. These reads were removed. Variant calling with the same filters of 1% frequency and SNP quality >1,000 similarly removed all false positives, and all 57 SNPs were called.

We aligned full sequences (BAsE-Seq) or multi-sequence aligned CCSs (Pacbio) against the reference genotype C, and constructed maximum likelihood trees. Both BAsE-Seq (SI Figure 6)

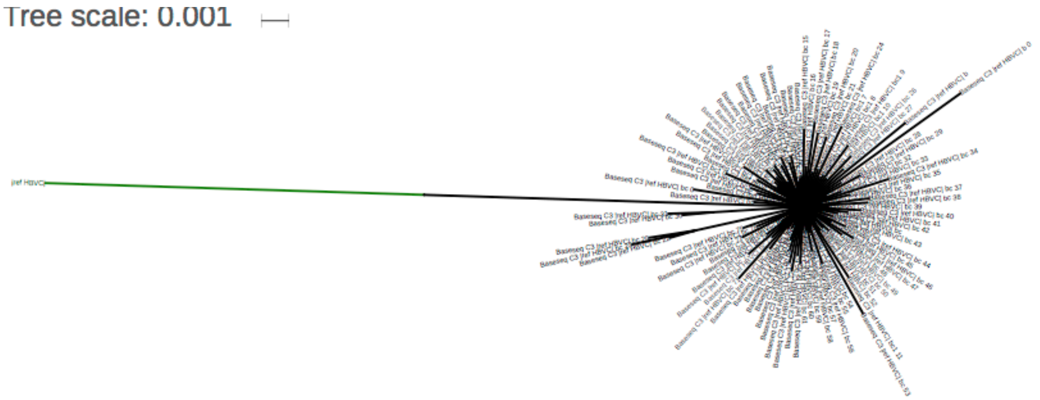

SI Figure 6. Clone2 library. Phylogenetic tree of full-length single virion sequences constructed from BAsE-Seq rooted at reference Genotype C (green branch). Each individual branch is labeled at the tips; only black solid lines represent branch length (nucleotide substitution per site).

and Pacbio (SI Figure 7)

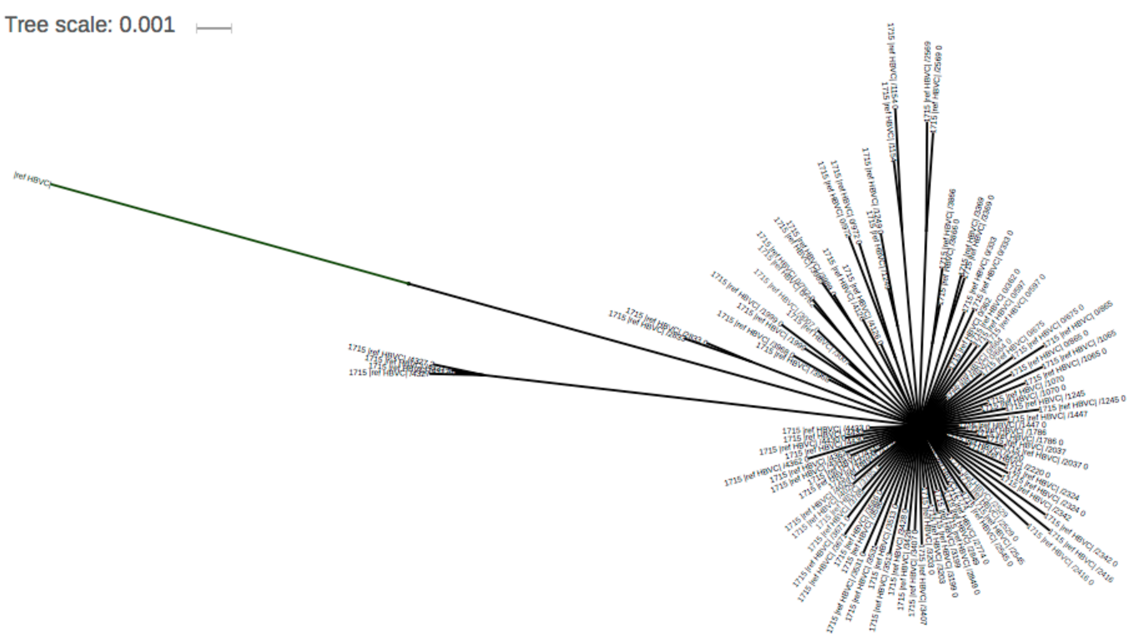

SI Figure 7. Clone2 library. Phylogenetic tree of full-length single virion sequences constructed from PacBio rooted at reference Genotype C (green branch). Each individual branch is labeled at the tips; only black solid lines represent branch length.

gave a single cluster of sequences a similar distance away from the reference. The longer branches on the Pacbio tree are due to multiple alignment artifacts from deletion prone Pacbio reads (SI Figure 3).

Clone-1:Clone-2 mixed population. Viral populations within a patient can be diverse. We sequenced a 50:50 mixture of two strains Clone-1 and Clone-2 to test the sensitivity of each method to a mixed population.

Illumina deep sequencing showed an average coverage depth of 30,000 within the reference genotype C sequence. Two dips in coverage were observed where deletions are present in either clone (SI Figure 8).

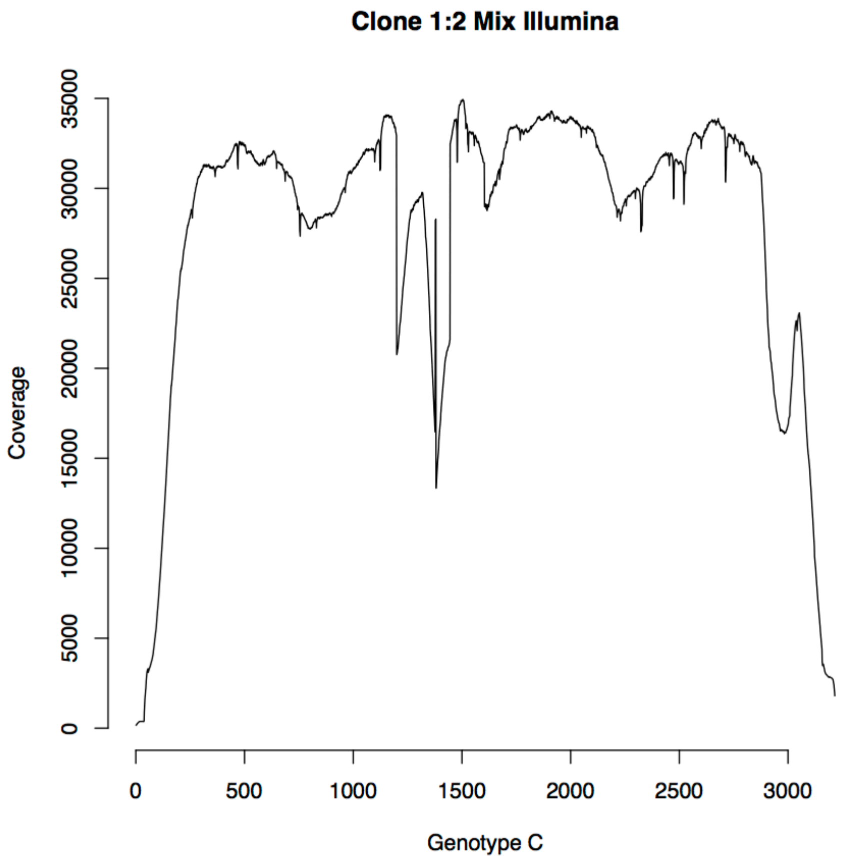

SI Figure 8. Coverage (Y-axis) plot against Genotype C (X-axis) for Clone 1:2 mix Pooled deep sequencing library. The first 40bp were not covered due to primer location, and two dips are seen due to deletions present on each clone – a 56bp deletion at position 1197 for Clone 1 and a 66bp deletion at position 1380 for Clone 2.

In Pooled deep sequencing libraries, SNPs were called for all bases covered to >5% average genomic coverage within the Genotype C sequence. Of the 65 SNPs expected, all were identified post coverage, quality, frequency, and strand bias filtering.

2,151 BAsE-Seq sequences passed filtering (>4x coverage depth at >1,500 bases). Of the 65 SNPs expected, all were identified post coverage, quality, frequency, and strand bias filtering.

6,299 10x CCSs from a single PacBio run were mapped as extremely long reads to the reference panel. 6,295 reads (>99.9%) mapped to the genotype C reference. Applying the same filters of 1% frequency and SNP quality >1,000, all 65 expected SNPs were identified.

All 17 known SNP differences between the clones registered at allele frequencies of 0.4-0.6 (SI Figure 9).

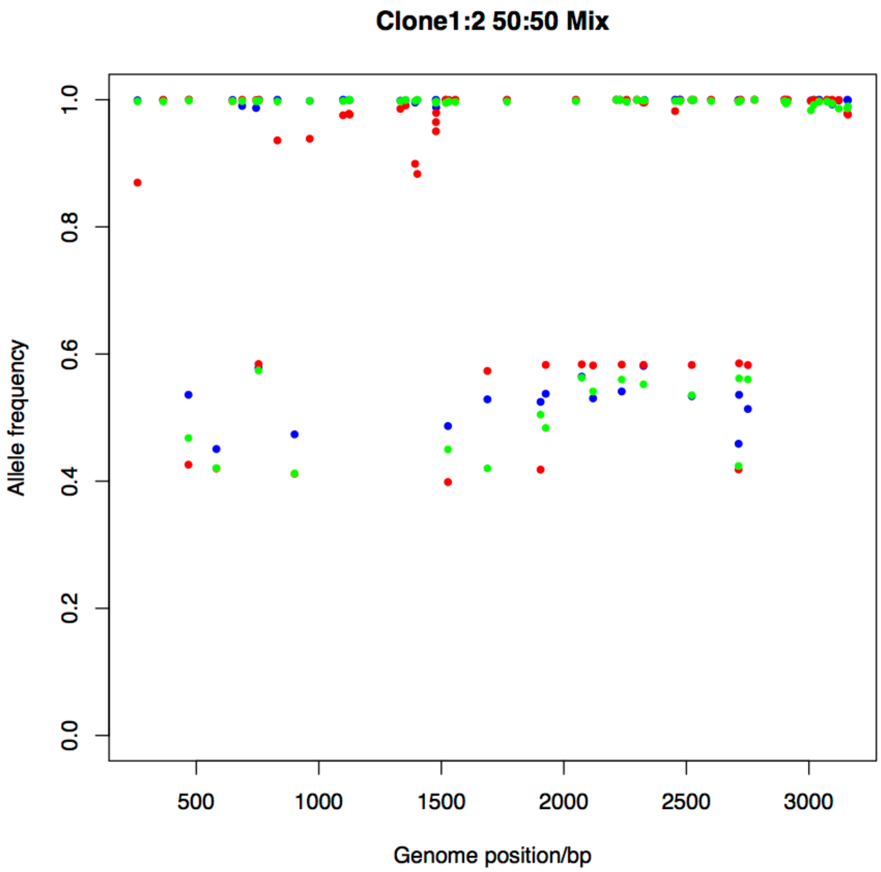

SI Figure 9. Allele frequency (Y-axis) plotted against Genotype C (X-axis) for Clone 1:2 mixed library. Frequencies from the platforms are represented as Red:BAsE-Seq, Green:Pooled deep sequencing, Blue:PacBio.

Both platforms performed well on accurately representing population structure, again with BAsE-Seq sequences (SI Figure 10)

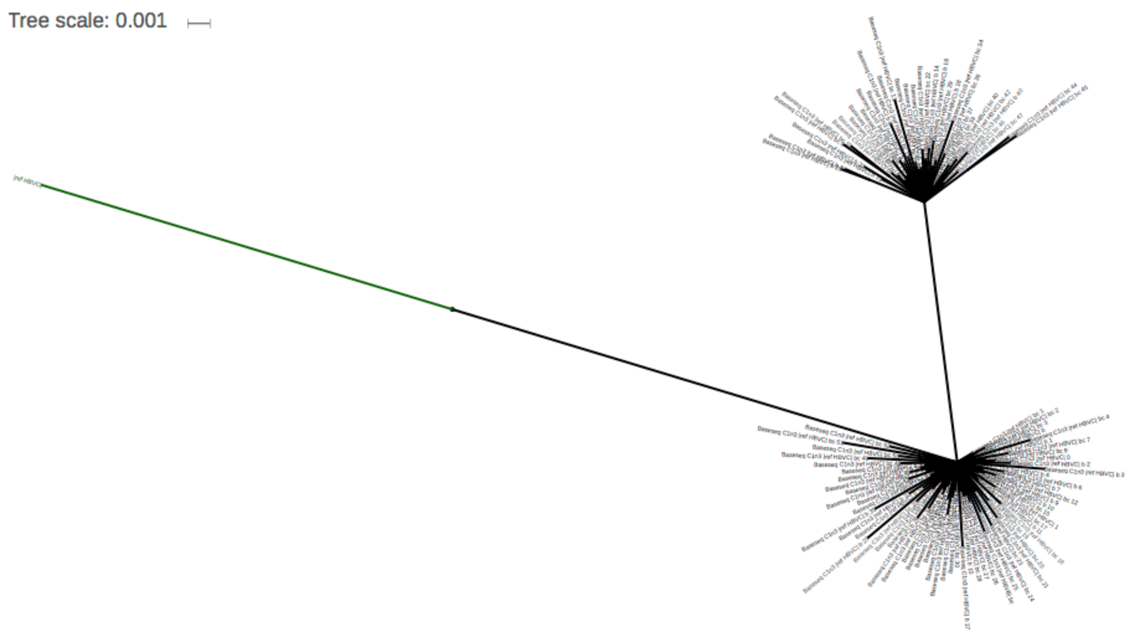

SI Figure 10. Clone1:2 mixed library. Phylogenetic tree of full-length single virion sequences constructed from BAsE-Seq rooted at reference Genotype C (green branch). Each individual branch is labeled at the tips; only black solid lines represent branch length.

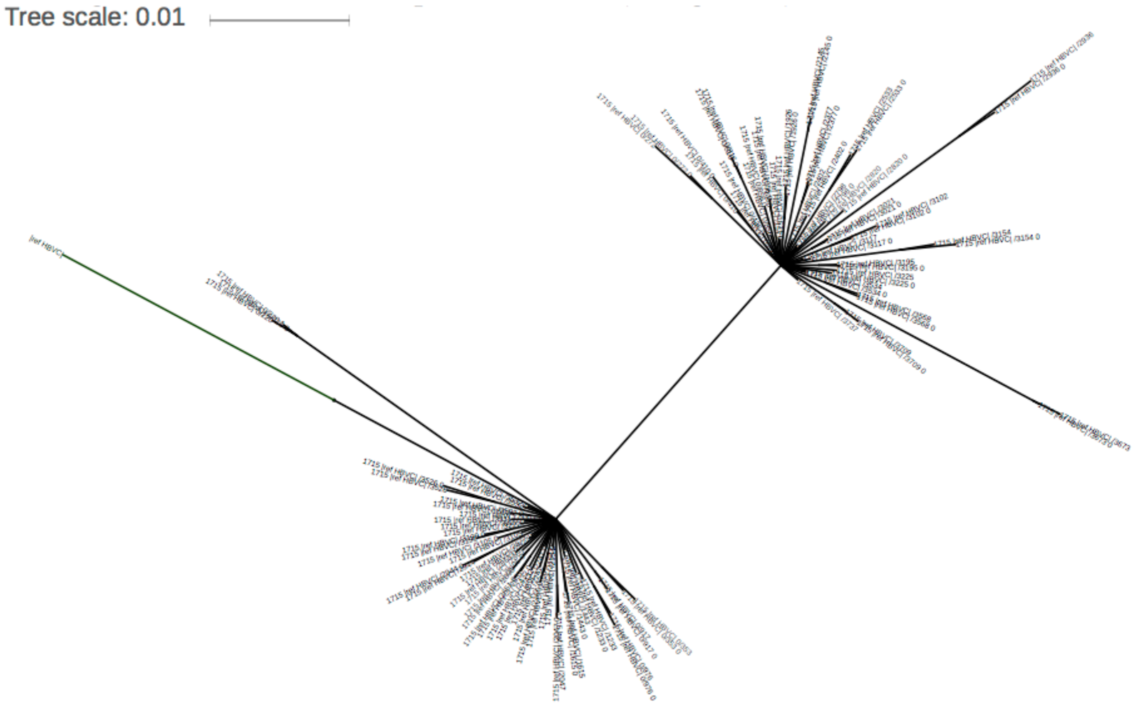

showing shorter branches as compared to Pacbio (SI Figure 11).

SI Figure 11. Clone1:2 mixed library. Phylogenetic tree of full-length single virion sequences constructed from BAsE-Seq rooted at reference Genotype C (green branch). Each individual branch is labeled at the tips; only black solid lines represent branch length.

Clinical Sample Viral Populations Viral genotype holds clinical implications. We tested the ability of each platform to accurately call and identify the dominant genotype in a patient, as well as mixed co-infections, if any. For samples Clone2, Clone1:Clone2 mix, P1.1, P1.2, P2.1, and P2.2, all three platforms identified the same dominant genotype for each sample (SI Figure 12).

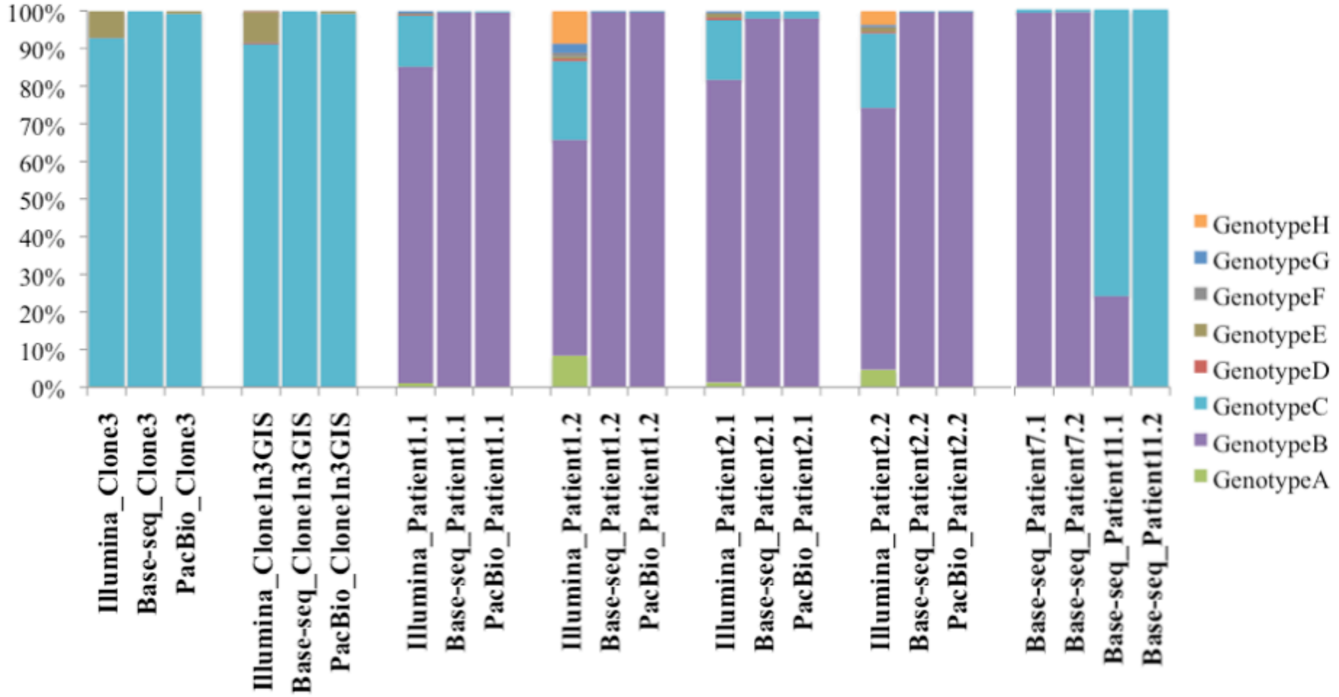

SI Figure 12. Genotype composition (Y-axis) of samples sequenced (X-axis) as reported by bwa-sw coverage across 8 reference genotype sequences. Samples are lined from left to right in order of Clone2, Clone1:2 mix, P1.1, P1.2, P2.1, P2.2, P7.1, P7.2, P11.1, P11.2. Wherever data from multiple technologies are available, order follows Pooled deep sequencing (Illumina), BAsE-Seq, and lastly PacBio.

Pooled deep sequencing short reads had higher mis-mapping rates that led to substantial coverage outside of the true genotype (up to 43% such as in P1.2), whereas long reads in BAsE-Seq and Pacbio gave nearly no mis-mapping. The low frequencies observed above (0%-0.3% for BAsE-Seq, 0.07%-0.4%) came from spike in controls.

Summary

All three methods perform well when it comes to identifying SNP polymorphisms and their frequencies. With timepoint data, any of these methods can identify sharp increases in variants that may be under selection, and suggest linkage between SNVs that change in frequency together. However, Pooled deep sequencing does not have definitive proof of exact linkage patterns, which can be limiting in more complex populations with multiple adaptive haplotypes. One other limitation with Pooled deep sequencing concerns the identification of mixed genotypes. If multiple genotypes are present at significant percentages, coverage across the pan-genome will reveal the genotypes present, and LoFreq results can be filtered accordingly, such as with Patient11.1 where both genotypes are present at >30%. However, the same cannot be said of low frequency mixed infections, such as the controlled spike-ins, which cannot be reliably identified through

Illumina mapping, but require long haplotype information such as those from BAsE-Seq and PacBio single virion sequences.

BAsE-Seq and PacBio perform similarly in terms of the number of single virion sequences obtainable from each library. The number of sequences obtained from every library made is listed below (SI Figure 13). There is substantial variation from library to library within each method, largely determined by patient sample quality, viral count, and run quality.

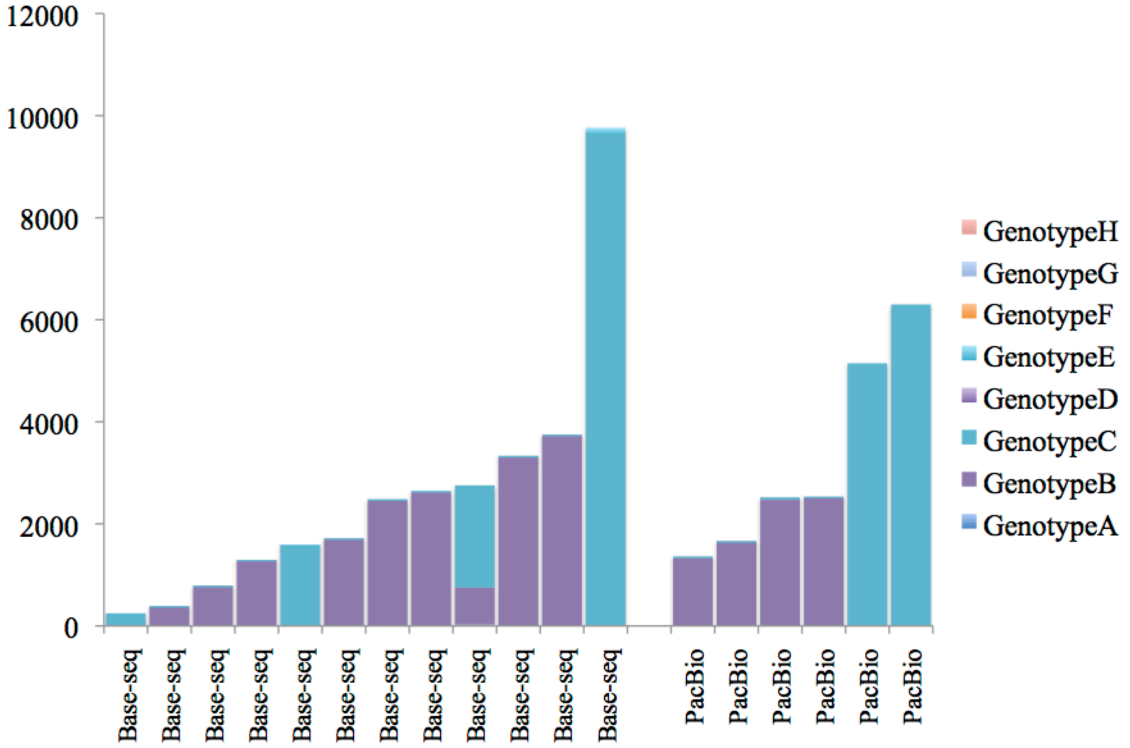

SI Figure 13. Number of haplotypes retrieved from each library, separated by sequencing platform, and sorted within each by count.

One limitation of BAsE-Seq is the absolute requirement of a reference genome. Whereas PacBio reads are more prone to small indel errors in homopolymer runs. Both methods run into some lengths limits. BAsE-Seq relies on a single PCR amplicon that can be barcoded and amplified. This becomes increasingly difficult to achieve beyond 5kb. Longer amplicons may also introduce more bias into the circularization step. PacBio relies on multiple reads of the same molecule. Given that the upper limit for a PacBio read was ~60kb at the time of this manuscript, and keeping in mind that the majority of the reads from a run will not be quite so long, the achievable haplotype length may not be significantly longer than Base-Seq. HBV genome is short and proves the value of such studies, and as technologies improve over time, larger and larger viral genomes can hopefully be explored.

Indels remain a tricky issue. Both BAsE-Seq (due to low coverage for individual bam files) and PacBio (due to error prone chemistry) will require specific experiments tailored to the development of indel calling pipelines. This falls outside of the scope of our manuscript

Full Phylogenetic Trees

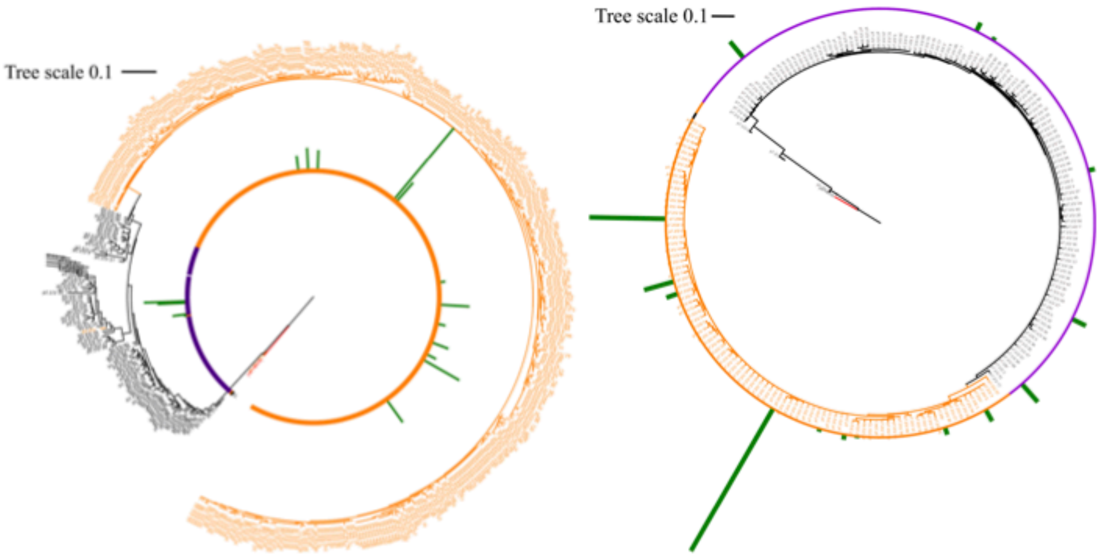

SI Figure 14,15. BAsE-Seq neighbor-joining trees of viral sequences from P2 (left) and P7 (right) before (black branches) and after (orange branches) drug resistance. Trees are rooted against reference genome HBV genotype B. Sequences from each timepoint are color-coded by their branch labels and the surrounding colored circle. Orange indicate sequences post resistance, black/purple indicate sequences pre-resistance. Green bars on the circle indicate abundance of each sequence where available.

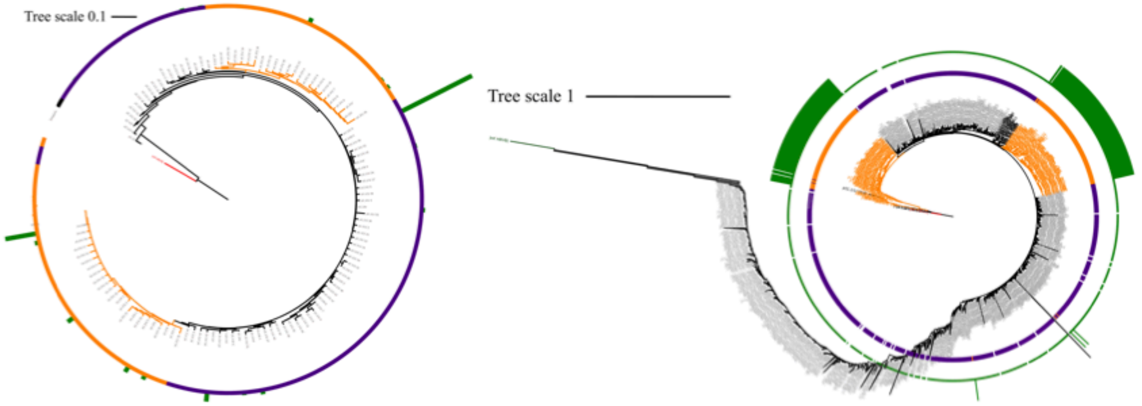

Figure 16,17. BAsE-Seq neighbor-joining tree of viral sequences from P1 (left) and P11 (right) before (black) and after (orange) drug resistance. Tree is rooted at reference genome HBV genotype B for P1 and genotype C for P11. Medium green circle surrounding the tree signifies abundance of each sequence type where available.

BEAST Bayesian Reconstruction of Population Demographic History

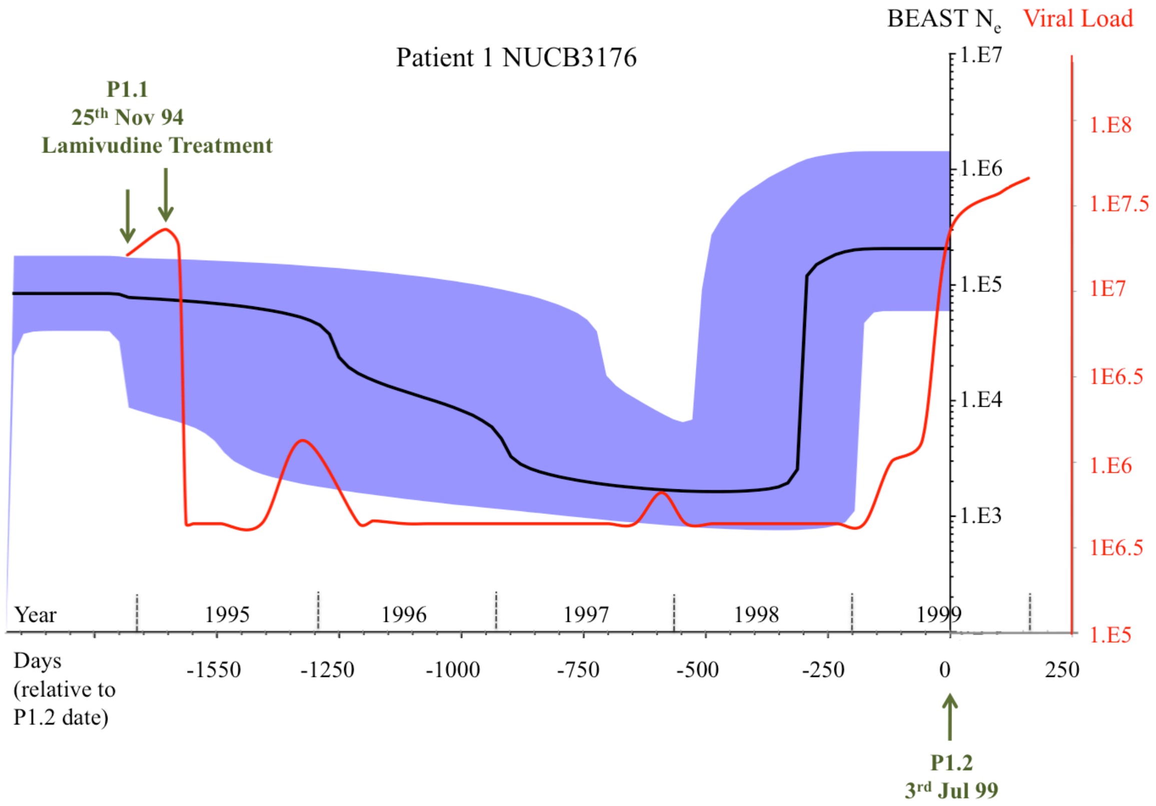

SI Figure 18. Overlay of BEAST reconstructed demographic history (changes in effective population size over time) and clinical records of patient viral load (serum) for patient 1. X-axis – Timeline represented in days, going forward in time from left to right. Green arrows point out the two timepoints that were used for single virion sequencing. Y-axis labeled "Viral load" – corresponding to red line tracing all available records of patient viral load (log10 scale) over time. Y-axis labeled "BEAST N_e" - Effective population size over time as simulated by BEAST (log10 scale) shown by black line (plotted values are local medians, with 95% highest density probability interval colored in blue).

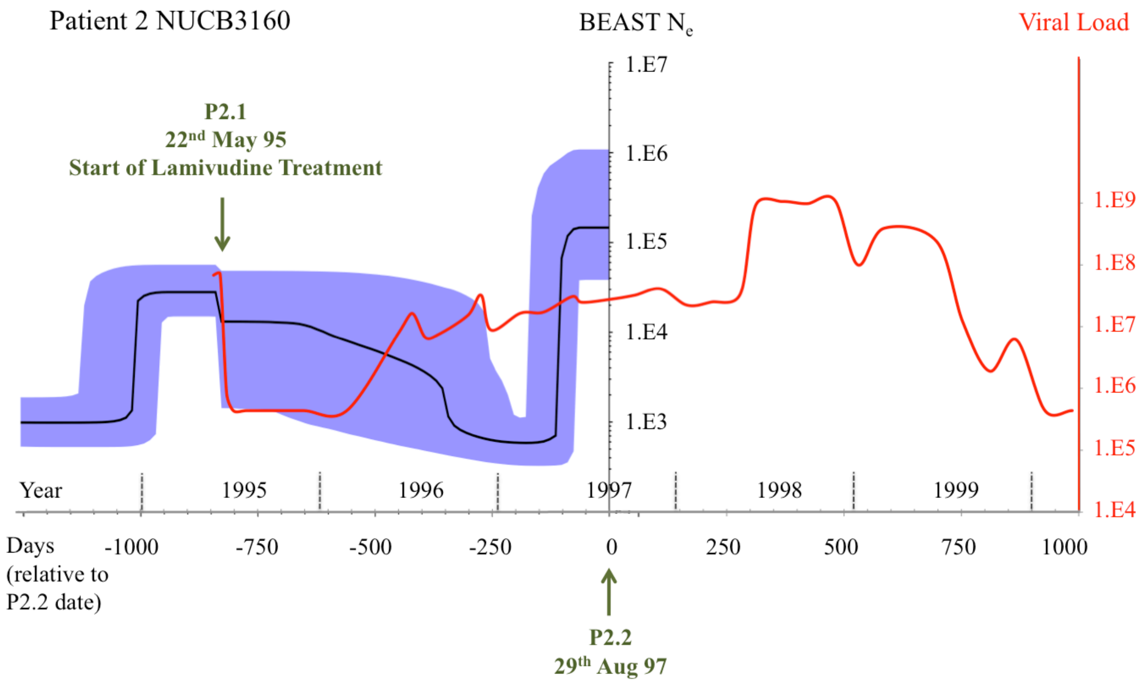

SI Figure 19. Overlay of BEAST reconstructed demographic history and clinical records of patient viral load (serum) for patient 2.

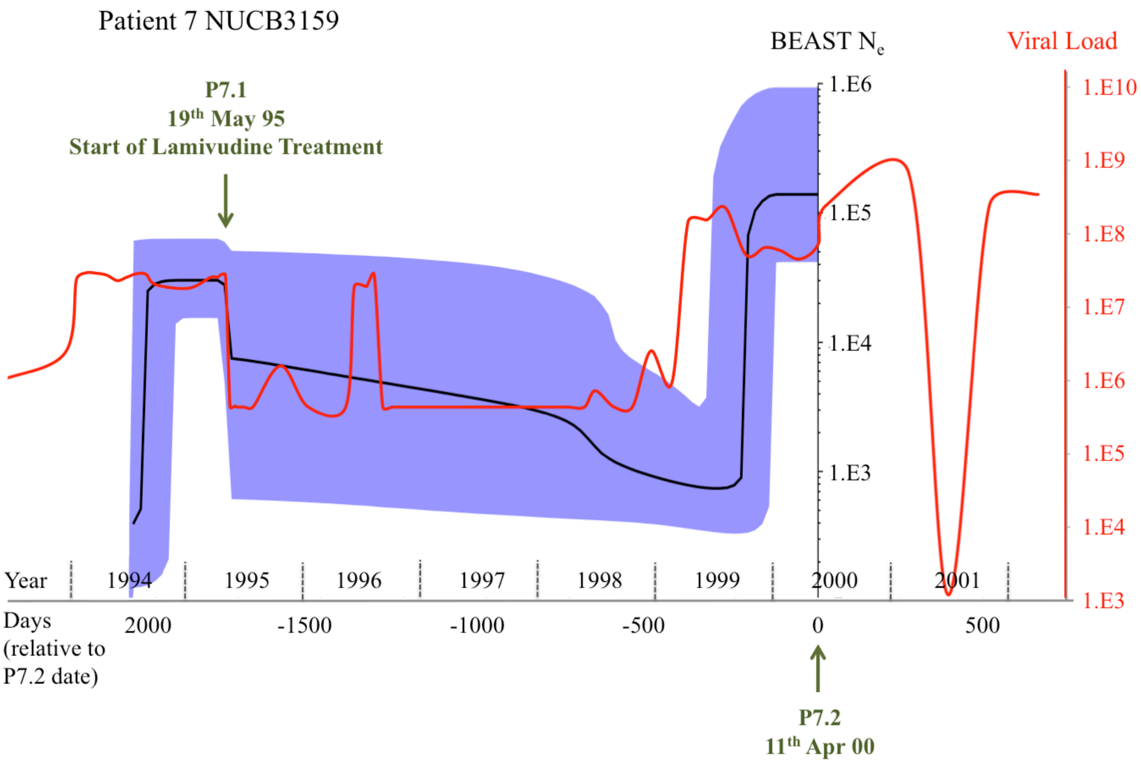

SI Figure 20. Overlay of BEAST reconstructed demographic history and clinical records of patient viral load (serum) for patient 7.

P1	HBV DNA copies
11-Dec-1999	45959200
16-Oct-1999	38035200
**3-Jul-1999**	**22187200**
8-May-1999	1346800
6-Mar-1999	1008000
9-Jan-1999	436800
14-Nov-1998	436800
26-Sep-1998	436800
25-Jul-1998	436800
6-Jun-1998	436800
11-Apr-1998	436800
5-Mar-1998	436800
10-Jan-1998	436800
21-Nov-1997	663600
27-Sep-1997	436800
2-Aug-1997	436800
7-Jun-1997	436800
12-Apr-1997	436800
15-Mar-1997	436800
18-Jan-1997	436800
23-Nov-1996	436800
28-Sep-1996	436800
3-Aug-1996	436800
15-Jun-1996	436800
10-Apr-1996	453600
12-Mar-1996	436800
18-Nov-1995	1341200
26-Aug-1995	436800
3-Jun-1995	436800
6-May-1995	436800
8-Apr-1995	436800
25-Mar-1995	436800
10-Mar-1995	18088000
11-Feb-1995	23066400
**25-Nov-1994**	**16296000**

P2	HBV DNA copies
11-May-2000	436800
14-Mar-2000	436800
13-Jan-2000	6101200
17-Nov-1999	1901200
21-Sep-1999	11886000
27-Jul-1999	240800000
6-Apr-1999	392000000
9-Feb-1999	100800000
22-Dec-1998	1120000000
24-Oct-1998	980000000
1-Sep-1998	1064000000
7-Jul-1998	924000000
4-Jun-1998	33941600
3-Apr-1998	25015200
10-Feb-1998	22316000
16-Dec-1997	41022800
21-Oct-1997	32197200
**29-Aug-1997**	**27568800**
1-Jul-1997	24752000
10-Jun-1997	30604000
10-Apr-1997	17192000
20-Feb-1997	16508800
19-Dec-1996	8811600
28-Nov-1996	32205600
31-Oct-1996	15288000
29-Aug-1996	7442400
1-Aug-1996	6680800
4-Jul-1996	16382800
5-Jun-1996	7859600
16-Feb-1996	436800
15-Nov-1995	436800
21-Aug-1995	436800
17-Jul-1995	436800
19-Jun-1995	436800
5-Jun-1995	817600
**22-May-1995**	**71612800**
8-May-1995	68311600

P7	HBV DNA copies
**11-Apr-2000**	**71316000**
15-Feb-2000	45166800
21-Dec-1999	58637600
3-Nov-1999	64960000
7-Sep-1999	51506000
6-Jul-1999	226800000
11-May-1999	154000000
16-Mar-1999	145600000
26-Jan-1999	924000
24-Nov-1998	2548000
5-Aug-1998	436800
9-Jun-1998	728000
20-Jan-1998	436800
30-Sep-1997	436800
20-Mar-1997	436800
5-Sep-1996	436800
10-Aug-1996	27678000
18-Jul-1996	19146400
11-Jun-1996	19098800
16-May-1996	436800
24-Jan-1996	436800
2-Nov-1995	1590400
10-Aug-1995	436800
2-Jun-1995	436800
**19-May-1995**	**27588400**
5-May-1995	27588400
25-Apr-1995	25572400
5-Apr-1995	25572400
27-Jan-1995	18118800
20-Oct-1994	20378400
22-Sep-1994	27977600
25-Aug-1994	28672000
27-Jun-1994	23055200
26-May-1994	27344800
25-Apr-1994	29041600
24-Mar-1994	29358000
24-Feb-1994	24136000
19-Jan-1994	2352000
30-Jun-1993	968800

P11	HBV DNA copies
28-Mar-2000	221872000
27-Jan-2000	237720000
7-Dec-1999	174328000
14-Oct-1999	221872000
19-Aug-1999	166404000
27-Apr-1999	134708000
23-Feb-1999	237720000
29-Dec-1998	158480000
**3-Nov-1998**	**158480000**
4-Sep-1998	142632000
14-Jul-1998	174328000
11-Jun-1998	7935200
24-Apr-1998	4740400
24-Feb-1998	9539600
6-Jan-1998	7537600
11-Nov-1997	4398800
9-Sep-1997	6081600
15-Jul-1997	4334400
17-Jun-1997	6988800
24-Apr-1997	7120400
27-Feb-1997	4799200
3-Jan-1997	1397200
7-Nov-1996	436800
12-Sep-1996	436800
18-Jul-1996	436800
19-Jun-1996	436800
1-Mar-1996	436800
9-Dec-1995	2553600
16-Sep-1995	436800
14-Aug-1995	436800
17-Jul-1995	436800
3-Jul-1995	826000
**19-Jun-1995**	**33860400**
9-Jun-1995	27232800

SI Table 3. Clinical records of patient viral load (serum) over time.
