# Supplementary material for: Single-virion sequencing of lamivudine-treated HBV populations reveal population evolution dynamics and demographic history
Source: BMC Genomics. 2017 Oct 27;18:829. doi: 10.1186/s12864-017-4217-1 (PMC5660452; doi:10.1186/s12864-017-4217-1)
Supplement: Supplementary file 3 — Supplementary_FastTrees.txt. Newick format phylogenetic trees. FastTree output in Newick format for all 4 patients (TXT 16 kb) [file 12864_2017_4217_MOESM3_ESM.txt]

P1(|ref_HBVB|:0.002815,(|ref_HBVC|:0.08159,(1.2_|ref_HBVB|_b_1:0.00214,((1.1_|ref_HBVB|_b:0.0,1.1_|ref_HBVC|_b:0.0):0.00084,((1.2_|ref_HBVB|_b_15:0.00071,1.1_|ref_HBVB|_bc:0.00066):0.00055[0.105],(1.1_|ref_HBVB|_b_15:0.00055,(1.1_|ref_HBVB|_bc_6:0.00055,(((1.1_|ref_HBVB|_bc_13:0.00085,1.1_|ref_HBVB|_bc_16:0.00055):0.00055[0.851],(((1.2_|ref_HBVB|_bc_3:0.00055,((((1.2_|ref_HBVB|_b_11:0.00055,(1.2_|ref_HBVB|_b_17:0.0,1.2_|ref_HBVB|_b_18:0.0,1.2_|ref_HBVB|_b_19:0.0,1.2_|ref_HBVF|_b:0.0):0.00055):0.00055[0.424],(1.2_|ref_HBVB|_bc_4:0.00055,(1.2_|ref_HBVB|_b_9:0.00055,1.2_|ref_HBVB|__0:0.00065):0.00055[0.531]):0.00055[1.000]):0.00055[0.853],(1.2_|ref_HBVB|_b_3:0.00054,1.2_|ref_HBVB|_bc:0.00055):0.00055[0.705]):0.00055[0.873],((((1.2_|ref_HBVB|_b_13:0.0,1.2_|ref_HBVC|_b:0.0):0.00097,(1.2_|ref_HBVB|_b_7:0.00055,1.2_|ref_HBVB|_bc_8:0.00089):0.00055[0.603]):0.00055[0.809],(1.2_|ref_HBVB|_b_4:0.00055,(1.2_|ref_HBVB|_b_14:0.0,1.2_|ref_HBVC|_b_0:0.0):0.00109):0.00055[0.799]):0.00055[0.917],((((1.2_|ref_HBVB|_bc_5:0.0,1.2_|ref_HBVC|_bc:0.0):0.00055,(1.2_|ref_HBVB|_bc_9:0.0,1.2_|ref_HBVB|_bc_10:0.0,1.2_|ref_HBVB|_bc_11:0.0):0.00055):0.00055[0.499],(1.2_|ref_HBVB|_b_0:0.00079,(((1.2_|ref_HBVB|_b_2:0.00055,1.2_|ref_HBVB|_b_25:0.00055):0.00055[0.383],(1.2_|ref_HBVB|_b_22:0.0,1.2_|ref_HBVB|_b_23:0.0):0.00055):0.00055[0.428],((1.2_|ref_HBVB|_bc_1:0.0,1.2_|ref_HBVB|_bc_2:0.0):0.00055,1.2_|ref_HBVB|_bc_0:0.00107):0.00055[0.641]):0.00055[0.800]):0.00055[0.732]):0.00055[0.992],((1.2_|ref_HBVB|_b_16:0.00118,(1.2_|ref_HBVB|_b_21:0.00055,(1.2_|ref_HBVB|_b:0.00055,(1.2_|ref_HBVB|_b_6:0.0,1.2_|ref_HBVB|_b_5:0.0):0.00077):0.00055[0.842]):0.00055[0.770]):0.00055[0.854],1.2_|ref_HBVB|_bc_6:0.00055):0.00055[0.954]):0.00055[0.736]):0.00055[0.995]):0.00055[0.271]):0.00083[0.910],(((1.1_|ref_HBVB|_b_2:0.00067,(1.1_|ref_HBVB|_bc_5:0.00055,(1.1_|ref_HBVB|_b_8:0.00075,(1.1_|ref_HBVB|_bc_10:0.0,1.1_|ref_HBVB|_bc_9:0.0):0.00156):0.00055[0.845]):0.00055[0.791]):0.00055[0.918],(((1.1_|ref_HBVB|_bc_17:0.0,1.1_|ref_HBVB|_bc_18:0.0,1.1_|ref_HBVB|_bc_19:0.0,1.1_|ref_HBVB|_bc_20:0.0):0.00055,(1.1_|ref_HBVB|_bc_12:0.00055,1.1_|ref_HBVB|_bc_22:0.00055):0.00055[0.533]):0.00055[0.948],(1.1_|ref_HBVB|_bc_4:0.00055,(1.1_|ref_HBVB|_bc_15:0.00051,(1.1_|ref_HBVB|_:0.00075,1.1_|ref_HBVB|_b_13:0.00054):0.00055[0.762]):0.00055[0.996]):0.00055[0.281]):0.00055[0.460]):0.00055[0.440],1.1_|ref_HBVB|_b_14:0.00055):0.00055[0.837]):0.00055[0.681],(1.2_|ref_HBVB|__1:0.00055,((1.2_|ref_HBVB|_b_8:0.00055,1.2_|ref_HBVB|_bc_7:0.00055):0.00077[0.879],(1.2_|ref_HBVB|_b_12:0.00055,((1.2_|ref_HBVB|_b_24:0.0,1.2_|ref_HBVC|_b_2:0.0,1.2_|ref_HBVC|_b_3:0.0):0.00055,1.2_|ref_HBVB|_b_10:0.00055):0.00055[1.000]):0.00055[0.268]):0.00092[0.916]):0.00055[0.853]):0.00055[0.738]):0.00055[0.154],(((1.1_|ref_HBVB|__0:0.00055,(1.2_|ref_HBVB|_b_20:0.0,1.2_|ref_HBVC|_b_1:0.0):0.00091):0.00055[0.945],((1.1_|ref_HBVB|_b_7:0.00055,1.2_|ref_HBVB|_:0.00096):0.00055[0.636],1.1_|ref_HBVB|_bc_7:0.00055):0.00055[0.993]):0.00055[0.999],((1.1_|ref_HBVB|_b_5:0.00113,(1.1_|ref_HBVB|_bc_0:0.00055,1.1_|ref_HBVB|_bc_8:0.00066):0.00055[0.280]):0.00055[0.895],((((1.1_|ref_HBVB|_bc_2:0.00055,(1.1_|ref_HBVB|_b_3:0.00084,1.1_|ref_HBVB|_b_10:0.00055):0.00055[0.852]):0.00055[0.986],(1.1_|ref_HBVB|_bc_3:0.00055,1.1_|ref_HBVB|_bc_14:0.00055):0.00055[0.853]):0.00055[0.910],(1.1_|ref_HBVB|_b_1:0.00065,1.1_|ref_HBVB|_bc_25:0.00065):0.00055[0.409]):0.00055[0.611],((1.1_|ref_HBVB|_b_9:0.00055,1.1_|ref_HBVB|_bc_24:0.00053):0.00055[0.855],((((((1.1_|ref_HBVB|_b_4:0.00055,1.1_|ref_HBVB|_b_12:0.00055):0.00055[0.984],1.1_|ref_HBVB|_b_0:0.00055):0.00055[0.000],(1.1_|ref_HBVB|_b_11:0.00065,(1.1_|ref_HBVB|_bc_1:0.00055,1.1_|ref_HBVB|_b_17:0.00055):0.00055[0.203]):0.00055[0.880]):0.00055[0.979],1.1_|ref_HBVB|_bc_11:0.00055):0.00055[0.498],(1.1_|ref_HBVB|_bc_21:0.00055,(1.1_|ref_HBVB|_bc_26:0.00055,1.1_|ref_HBVB|_b_6:0.00055):0.00055[0.000]):0.00055[0.740]):0.00055[0.545],(1.1_|ref_HBVB|_bc_23:0.00065,1.1_|ref_HBVB|_b_16:0.00065)OROOT:0.00055[0.321]):0.00055[0.932]):0.00055[0.838]):0.00055[0.790]):0.00055[0.191]):0.00055[0.483]):0.00055[0.837]):0.00055[0.989]):0.00055[1.000]):0.00055[0.763]):0.00055[0.414]):0.00640[0.988]):0.002815);P2(|ref_HBVB|:0.00169,(|ref_HBVC|:0.08288,(2.1_|ref_HBVB|_bc_22:0.00818,((2.1_|ref_HBVB|_bc_6:0.0,2.1_|ref_HBVB|_bc_7:0.0):0.00116,(2.1_|ref_HBVB|_bc_2:0.00054,(((2.1_|ref_HBVB|_bc_13:0.00103,(2.1_|ref_HBVB|_bc_8:0.00067,2.1_|ref_HBVB|_bc_34:0.00074):0.00066[0.915]):0.00055[0.092],((((2.1_|ref_HBVB|_bc:0.0,2.1_|ref_HBVB|_bc_0:0.0):0.00058,2.1_|ref_HBVB|_bc_1:0.00233):0.00174[0.956],((2.1_|ref_HBVB|_bc_23:0.0,2.1_|ref_HBVB|_bc_24:0.0,2.1_|ref_HBVB|_bc_25:0.0):0.00055,(2.1_|ref_HBVB|_bc_21:0.00185,((2.1_|ref_HBVB|_bc_3:0.0,2.1_|ref_HBVB|_bc_4:0.0):0.00055,(2.1_|ref_HBVB|_b_0:0.0,2.1_|ref_HBVB|_b_1:0.0,2.1_|ref_HBVF|_b:0.0):0.00055):0.00055[0.765]):0.00055[0.498]):0.00051[0.091]):0.00054[0.816],(2.1_|ref_HBVB|_b_2:0.0,2.1_|ref_HBVB|_b_3:0.0):0.00211):0.00054[0.964]):0.00051[0.029],((2.1_|ref_HBVB|_b:0.0,2.1_|ref_HBVC|_b:0.0):0.00070,(2.1_|ref_HBVB|_bc_30:0.00055,(2.1_|ref_HBVB|_bc_26:0.00055,((2.1_|ref_HBVB|_bc_27:0.00055,(2.1_|ref_HBVB|_b_6:0.00175,(((2.1_|ref_HBVB|_:0.0,2.1_|ref_HBVB|__0:0.0,2.1_|ref_HBVB|__1:0.0):0.00081,2.1_|ref_HBVB|_bc_10:0.00173):0.00055[0.841],((((2.1_|ref_HBVB|_bc_11:0.00082,((2.1_|ref_HBVB|_bc_31:0.0,2.1_|ref_HBVB|_bc_32:0.0,2.1_|ref_HBVB|_bc_33:0.0):0.00093,(2.1_|ref_HBVB|_bc_14:0.0,2.1_|ref_HBVB|_bc_15:0.0,2.1_|ref_HBVB|_bc_16:0.0,2.1_|ref_HBVB|_bc_17:0.0,2.1_|ref_HBVB|_bc_18:0.0,2.1_|ref_HBVE|_bc:0.0):0.00055):0.00055[0.364]):0.00055[0.365],(2.1_|ref_HBVB|_bc_28:0.00055,(2.1_|ref_HBVB|_b_4:0.0,2.1_|ref_HBVB|_b_5:0.0):0.00055):0.00055[0.786]):0.00055[0.596],((2.1_|ref_HBVB|_bc_9:0.00071,(2.1_|ref_HBVB|_bc_19:0.0,2.1_|ref_HBVB|_bc_20:0.0):0.00250):0.00055[0.844],2.1_|ref_HBVB|_bc_12:0.00069):0.00055[0.759]):0.00055[0.905],2.1_|ref_HBVB|_bc_5:0.00169):0.00071[0.910]):0.00143[0.961]):0.00110[0.921]):0.00055[0.465],(2.1_|ref_HBVB|_bc_29:0.00072,(2.2_|ref_HBVB|_:0.00103,(2.2_|ref_HBVB|_bc_27:0.00055,(2.2_|ref_HBVB|_bc_6:0.00055,((2.2_|ref_HBVB|_b:0.00055,(2.2_|ref_HBVB|_bc_35:0.00055,2.2_|ref_HBVB|_bc_28:0.00131):0.00055[0.999]):0.00055[1.000],((((2.2_|ref_HBVB|_bc:0.00054,((2.2_|ref_HBVB|_b_5:0.00055,(2.2_|ref_HBVB|_bc_10:0.00055,(2.2_|ref_HBVB|_bc_8:0.00055,2.2_|ref_HBVB|_bc_22:0.00097):0.00055[0.737]):0.00055[0.916]):0.00055[0.997],((2.2_|ref_HBVB|_bc_24:0.0,2.2_|ref_HBVB|_bc_26:0.0,2.2_|ref_HBVB|_bc_25:0.0):0.00145,2.2_|ref_HBVB|_bc_3:0.00068):0.00055[0.907]):0.00055[0.855]):0.00055[0.267],(2.2_|ref_HBVB|_b_4:0.00055,(2.2_|ref_HBVB|_bc_9:0.00055,(2.2_|ref_HBVB|_bc_31:0.0,2.2_|ref_HBVB|_bc_32:0.0,2.2_|ref_HBVB|_bc_33:0.0,2.2_|ref_HBVB|_bc_34:0.0):0.00055):0.00066[0.897]):0.00055[0.836]):0.00055[0.753],(((2.2_|ref_HBVB|_bc_4:0.0,2.2_|ref_HBVB|_bc_5:0.0):0.00055,(2.2_|ref_HBVB|_bc_12:0.0,2.2_|ref_HBVB|_bc_13:0.0,2.2_|ref_HBVB|_bc_14:0.0,2.2_|ref_HBVB|_bc_15:0.0):0.00055):0.00055[0.795],((2.2_|ref_HBVB|_bc_20:0.0,2.2_|ref_HBVB|_bc_21:0.0,2.2_|ref_HBVC|_bc:0.0,2.2_|ref_HBVC|_bc_0:0.0):0.00074,2.2_|ref_HBVB|_bc_30:0.00068):0.00055[0.292]):0.00055[0.920]):0.00055[0.987],(((2.2_|ref_HBVB|_bc_36:0.00116,(2.2_|ref_HBVB|_bc_1:0.00055,((2.2_|ref_HBVB|_bc_37:0.00073,(2.2_|ref_HBVB|_bc_18:0.0,2.2_|ref_HBVB|_bc_19:0.0):0.00210):0.00053[0.916],(2.2_|ref_HBVB|_bc_7:0.00066,(2.2_|ref_HBVB|_bc_11:0.00066,2.2_|ref_HBVB|_b_3:0.00066):0.00055[0.528]):0.00055[0.888]):0.00055[0.402]):0.00055[0.779]):0.00055[0.427],(2.2_|ref_HBVB|_bc_16:0.00055,2.2_|ref_HBVB|_bc_23:0.00065):0.00055[0.918]):0.00055[0.865],(((2.2_|ref_HBVB|_b_1:0.0,2.2_|ref_HBVB|_b_2:0.0):0.00055,2.2_|ref_HBVB|_bc_38:0.00097):0.00055[0.840],(2.2_|ref_HBVB|_bc_29:0.00055,((2.2_|ref_HBVB|_bc_0:0.00055,2.2_|ref_HBVB|_b_0:0.00055):0.00055[0.735],(2.2_|ref_HBVB|_bc_17:0.00097,2.2_|ref_HBVB|_bc_2:0.00055):0.00055[0.248]):0.00055[0.996])OROOT:0.00055[0.984]):0.00055[0.962]):0.00055[0.444]):0.00055[0.750]):0.00055[0.854]):0.00055[0.859]):0.00064[0.327]):0.00315[0.993]):0.00055[0.847]):0.00055[0.273]):0.00055[0.694]):0.00055[0.822]):0.00055[0.904]):0.00055[0.785]):0.00051[0.879]):0.00472[0.993]):0.00259[0.444]):0.00169);P7(|ref_HBVB|:0.00174,(7.2_|ref_HBVB|__1:0.00416,(7.2_|ref_HBVB|_b_16:0.00057,(7.1_|ref_HBVB|_bc_6:0.00055,((7.1_|ref_HBVB|__0:0.00055,((((7.1_|ref_HBVB|_bc_8:0.00055,(7.1_|ref_HBVB|_bc_24:0.00055,7.1_|ref_HBVB|_b_10:0.00055):0.00055[0.870]):0.00055[0.919],(((((7.1_|ref_HBVB|_b_11:0.00065,7.1_|ref_HBVB|_:0.00055):0.00055[0.878],(7.1_|ref_HBVB|_bc_9:0.00066,7.1_|ref_HBVB|_bc_26:0.00055):0.00055[0.973]):0.00055[0.895],(((7.1_|ref_HBVB|_bc_3:0.00055,(7.1_|ref_HBVB|_b_2:0.00055,7.1_|ref_HBVB|_bc_20:0.00055):0.00055[0.739]):0.00055[0.917],(7.1_|ref_HBVB|_bc_1:0.00066,((((7.1_|ref_HBVB|_bc_17:0.00055,(7.1_|ref_HBVB|_bc_19:0.00055,7.1_|ref_HBVB|_b_7:0.00074):0.00055[0.902]):0.00055[0.735],7.1_|ref_HBVB|_bc_11:0.00055):0.00055[0.839],7.1_|ref_HBVB|_bc_4:0.00066):0.00055[1.000],(7.1_|ref_HBVB|_b_9:0.00055,((7.1_|ref_HBVB|_bc_16:0.00066,7.1_|ref_HBVB|_bc_27:0.00065):0.00055[0.839],(7.1_|ref_HBVB|_bc_2:0.00098,7.1_|ref_HBVB|_b_8:0.00055):0.00055[0.999]):0.00055[0.734]):0.00055[0.733]):0.00055[0.794]):0.00055[0.000]):0.00055[0.994],(7.1_|ref_HBVB|_bc:0.00055,7.1_|ref_HBVB|_bc_13:0.00119):0.00055[0.841]):0.00055[0.448]):0.00055[0.956],(7.1_|ref_HBVB|_bc_22:0.00055,(7.1_|ref_HBVB|_bc_12:0.00055,7.1_|ref_HBVB|_b_0:0.00071):0.00055[1.000]):0.00055[0.882]):0.00055[1.000],((7.1_|ref_HBVB|_b_1:0.00055,((7.1_|ref_HBVB|_bc_25:0.00055,(7.1_|ref_HBVB|_bc_0:0.00055,7.1_|ref_HBVB|_bc_7:0.00067):0.00055[0.223]):0.00055[0.738],(7.1_|ref_HBVB|_bc_21:0.00099,(7.1_|ref_HBVB|_bc_18:0.00055,7.1_|ref_HBVB|_b_6:0.00066):0.00055[0.833]):0.00055[0.988]):0.00055[0.913]):0.00055[0.800],(7.1_|ref_HBVB|_bc_5:0.00055,(7.1_|ref_HBVB|_b_3:0.0,7.1_|ref_HBVB|_b_4:0.0,7.1_|ref_HBVB|_b_5:0.0):0.00082):0.00055[0.338]):0.00055[0.765]):0.00055[0.737]):0.00055[0.703],((7.1_|ref_HBVB|_bc_15:0.00066,7.1_|ref_HBVB|_bc_23:0.00055):0.00055[0.738],(7.1_|ref_HBVB|_bc_14:0.00055,(7.1_|ref_HBVB|_bc_28:0.0,7.1_|ref_HBVC|_bc:0.0):0.00055):0.00055[0.911]):0.00055[0.939]):0.00055[0.691],7.1_|ref_HBVB|_bc_10:0.00055):0.00055[0.000]):0.00055[0.831],((7.2_|ref_HBVB|_bc:0.00137,7.2_|ref_HBVB|_b_32:0.00053):0.00050[0.424],(7.2_|ref_HBVB|_b_31:0.00055,(((7.2_|ref_HBVB|_b_7:0.00054,7.2_|ref_HBVB|_b_29:0.00074):0.00055[0.991],(7.2_|ref_HBVB|_b_23:0.00055,7.2_|ref_HBVB|__2:0.00055):0.00055[0.919]):0.00055[0.919],((7.2_|ref_HBVB|_b_8:0.00055,((7.2_|ref_HBVB|_b_5:0.00055,((7.2_|ref_HBVB|_b_34:0.00055,7.2_|ref_HBVB|_b_42:0.00055):0.00055[0.944],7.2_|ref_HBVB|_b_12:0.00055):0.00055[0.992]):0.00055[0.710],7.2_|ref_HBVB|_b_6:0.00065):0.00055[0.999]):0.00055[0.919],(((7.2_|ref_HBVB|_b_26:0.00055,(7.2_|ref_HBVB|_b_43:0.00065,(7.2_|ref_HBVB|_b_27:0.00055,(7.2_|ref_HBVB|_b_4:0.00065,(7.2_|ref_HBVB|_b_10:0.00065,7.2_|ref_HBVB|_b_35:0.00071):0.00055[0.000]):0.00055[0.000]):0.00055[0.449]):0.00055[0.585]):0.00055[0.753],(7.2_|ref_HBVB|_b_13:0.00065,((7.2_|ref_HBVB|_b_11:0.00055,7.2_|ref_HBVB|_b_28:0.00055):0.00055[0.734],(7.2_|ref_HBVB|_:0.00055,7.2_|ref_HBVB|_b_38:0.00055):0.00055[0.997]):0.00055[0.407]):0.00055[0.912]):0.00055[1.000],(((((7.2_|ref_HBVB|_b_40:0.00055,((7.2_|ref_HBVB|_b_0:0.00055,7.2_|ref_HBVB|_b_2:0.00055):0.00055[0.465],7.2_|ref_HBVB|_b_19:0.00097):0.00055[0.739]):0.00055[0.767],((7.2_|ref_HBVB|_b_22:0.00121,(7.2_|ref_HBVB|_b_17:0.00055,7.2_|ref_HBVB|_b_41:0.00195):0.00055[0.750]):0.00055[0.850],7.2_|ref_HBVB|_b_3:0.00055):0.00055[0.779]):0.00055[0.947],(7.2_|ref_HBVB|_b_30:0.00086,(7.2_|ref_HBVB|_b_9:0.00052,(7.2_|ref_HBVB|_b:0.00206,7.2_|ref_HBVB|_b_14:0.00055):0.00055[0.946]):0.00055[0.418]):0.00055[0.848]):0.00055[0.563],(7.2_|ref_HBVB|_b_15:0.00055,(7.2_|ref_HBVB|_b_24:0.00065,7.2_|ref_HBVB|_b_37:0.00055):0.00055[0.295]):0.00055[0.885]):0.00055[0.952],((7.2_|ref_HBVB|_b_18:0.00055,7.2_|ref_HBVB|_b_39:0.00055):0.00055[0.764],(7.2_|ref_HBVB|_b_36:0.00055,(7.2_|ref_HBVB|_b_21:0.00055,((7.2_|ref_HBVB|__0:0.00065,7.2_|ref_HBVB|_b_20:0.00065):0.00055[0.861],7.2_|ref_HBVB|_b_25:0.00064):0.00055[0.742]):0.00055[0.000]):0.00055[0.635]):0.00055[0.848])OROOT:0.00055[0.605]):0.00055[0.732]):0.00055[0.412]):0.00055[0.393]):0.00055[0.804]):0.00099[0.948]):0.00055[0.798]):0.00101[0.848]):0.00323[0.951]):0.00300[0.973]);P11(|ref_HBVB|:0.00551,(11.1_|ref_HBVB|_bc_0:0.00064,(11.1_|ref_HBVB|_bc:0.00055,((11.1_|ref_HBVB|_b_0:0.00055,(11.1_|ref_HBVB|_b_1:0.0,11.1_|ref_HBVC|_b_8:0.0,11.1_|ref_HBVC|_b_9:0.0,11.1_|ref_HBVC|_b_10:0.0):0.00055):0.00055[0.818],((11.1_|ref_HBVB|_bc_1:0.0,11.1_|ref_HBVB|_bc_2:0.0,11.1_|ref_HBVC|_bc_20:0.0):0.00053,((11.1_|ref_HBVB|_b:0.0,11.1_|ref_HBVC|_b:0.0,11.1_|ref_HBVC|_b_0:0.0):0.00054,(((11.1_|ref_HBVC|_bc_6:0.00278,(11.1_|ref_HBVC|_bc_9:0.00113,((11.1_|ref_HBVC|_bc_7:0.0,11.1_|ref_HBVC|_bc_8:0.0):0.00190,(11.1_|ref_HBVC|_:0.0,11.1_|ref_HBVC|__0:0.0,11.1_|ref_HBVC|__1:0.0):0.00277):0.00054[0.745]):0.00072[0.886]):0.00055[0.792],((11.1_|ref_HBVC|_b_4:0.00097,(11.2_|ref_HBVC|_b_40:0.00261,(11.1_|ref_HBVC|_bc_1:0.00075,11.1_|ref_HBVC|_bc_10:0.00211):0.00055[0.242]):0.00139[0.871]):0.00206[1.000],((((11.1_|ref_HBVC|_bc_19:0.00286,11.1_|ref_HBVC|_b_5:0.00175):0.00055[0.674],(11.1_|ref_HBVC|_bc:0.0,11.1_|ref_HBVC|_bc_0:0.0,11.1_|ref_HBVD|_bc:0.0):0.00121):0.00145[0.806],((11.1_|ref_HBVC|_bc_14:0.0,11.1_|ref_HBVC|_bc_15:0.0):0.00094,(11.1_|ref_HBVC|_bc_5:0.00500,(11.1_|ref_HBVC|_b_2:0.0,11.1_|ref_HBVC|_b_3:0.0):0.00055):0.00310[0.990]):0.00252[0.971]):0.00055[0.781],((11.1_|ref_HBVC|_b_6:0.0,11.1_|ref_HBVC|_b_7:0.0):0.00189,(((11.1_|ref_HBVC|__2:0.0,11.1_|ref_HBVC|__3:0.0,11.1_|ref_HBVC|__4:0.0):0.00281,((11.1_|ref_HBVC|_bc_2:0.0,11.1_|ref_HBVC|_bc_3:0.0,11.1_|ref_HBVC|_bc_4:0.0):0.00482,(11.1_|ref_HBVC|_bc_21:0.0,11.1_|ref_HBVC|_bc_22:0.0):0.00279):0.00116[0.586]):0.00153[0.933],((11.1_|ref_HBVC|_bc_11:0.0,11.1_|ref_HBVC|_bc_12:0.0,11.1_|ref_HBVC|_bc_13:0.0):0.00055,(11.1_|ref_HBVC|_bc_16:0.0,11.1_|ref_HBVC|_bc_17:0.0,11.1_|ref_HBVC|_bc_18:0.0):0.00247):0.00060[0.868]):0.00192[0.977]):0.00055[0.770]):0.00055[0.827]):0.00053[0.836]):0.00054[0.186],(((11.2_|ref_HBVC|_b_24:0.0,11.2_|ref_HBVC|_b_25:0.0,11.2_|ref_HBVC|_b_26:0.0,11.2_|ref_HBVC|_b_27:0.0):0.00059,(|ref_HBVC|:0.01214,(11.2_|ref_HBVC|_b_32:0.00139,(11.2_|ref_HBVC|_b_6:0.00118,11.2_|ref_HBVC|_b_0:0.00055):0.00053[0.536]):0.00859[1.000]):0.01344[1.000]):0.00926[1.000],((((((11.2_|ref_HBVC|__3:0.0,11.2_|ref_HBVC|__4:0.0,11.2_|ref_HBVC|__5:0.0,11.2_|ref_HBVC|__6:0.0):0.00132,(11.2_|ref_HBVC|_b_8:0.00170,11.2_|ref_HBVC|_b_19:0.00055):0.00053[0.572]):0.00054[0.987],((11.2_|ref_HBVC|_b_28:0.00054,(11.1_|ref_HBVC|_b_1:0.00503,11.2_|ref_HBVC|_b_22:0.00188):0.00339[0.993]):0.00055[0.058],((((11.2_|ref_HBVC|__0:0.00125,(11.2_|ref_HBVC|_b:0.00107,11.2_|ref_HBVC|_b_20:0.00169):0.00086[0.904]):0.00052[0.165],11.2_|ref_HBVC|_b_14:0.00179):0.00055[0.911],(11.2_|ref_HBVC|_b_16:0.00055,11.2_|ref_HBVC|_b_33:0.00310):0.00055[0.879]):0.00055[0.772],11.2_|ref_HBVC|_:0.00130):0.00055[0.838]):0.00054[0.754]):0.00055[0.738],(11.2_|ref_HBVC|_b_18:0.00082,11.2_|ref_HBVC|_b_9:0.00125):0.00066[0.753]):0.00055[0.750],(11.2_|ref_HBVC|_b_34:0.00107,11.2_|ref_HBVC|_b_5:0.00055):0.00053[0.961]):0.00159[0.978],(((((11.2_|ref_HBVC|_b_3:0.0,11.2_|ref_HBVC|_b_4:0.0):0.00085,11.2_|ref_HBVC|__1:0.00240):0.00093[0.873],((11.2_|ref_HBVC|_b_10:0.0,11.2_|ref_HBVC|_b_11:0.0):0.00055,11.2_|ref_HBVC|_b_35:0.00349):0.00096[0.941]):0.00055[0.743],11.2_|ref_HBVC|_b_12:0.00253):0.00054[0.969],((11.2_|ref_HBVC|_b_13:0.00052,(11.2_|ref_HBVC|_b_37:0.0,11.2_|ref_HBVC|_b_38:0.0):0.00055):0.00055[0.651],(11.2_|ref_HBVC|__2:0.00144,((11.2_|ref_HBVC|_b_1:0.00055,(11.2_|ref_HBVC|_b_2:0.00097,11.2_|ref_HBVC|_b_7:0.00078):0.00055[0.245]):0.00055[0.920],((11.2_|ref_HBVC|_b_15:0.00083,(11.2_|ref_HBVC|_b_21:0.00065,(11.2_|ref_HBVC|_b_36:0.00055,(11.2_|ref_HBVC|_b_17:0.00092,11.2_|ref_HBVC|_b_31:0.00135):0.00055[0.177]):0.00070[0.897]):0.00055[0.856]):0.00065[0.781],((11.2_|ref_HBVC|_b_29:0.00055,11.2_|ref_HBVC|_b_30:0.00055):0.00055[0.947],(11.2_|ref_HBVC|_b_23:0.00055,11.2_|ref_HBVC|_b_39:0.00096)OROOT:0.00055[0.991]):0.00055[0.913]):0.00055[0.740]):0.00055[0.789]):0.00055[0.850]):0.00055[0.892]):0.00063[0.888]):0.00086[0.905]):0.00263[0.984]):0.02253[1.000]):0.02692[1.000]):0.00398[0.998]):0.00220[0.983]):0.01348[1.000]):0.00551);
